# A Nitrogen-specific Interactome Analysis Sheds Light on the Role of the SnRK1 and TOR Kinases in Plant Nitrogen Signaling

**DOI:** 10.1016/j.mcpro.2024.100842

**Published:** 2024-09-20

**Authors:** Freya Persyn, Wouter Smagghe, Dominique Eeckhout, Toon Mertens, Thomas Smorscek, Nancy De Winne, Geert Persiau, Eveline Van De Slijke, Nathalie Crepin, Astrid Gadeyne, Jelle Van Leene, Geert De Jaeger

**Affiliations:** 1 Department of Plant Biotechnology and Bioinformatics, Ghent University, Ghent, Belgium; 2 VIB Center for Plant Systems Biology, Ghent, Belgium

**Keywords:** nitrogen signaling, Snf-related protein kinase 1 (SnRK1), target of rapamycin (TOR), *Arabidopsis thaliana*, protein interactome, AP-MS, proximity labeling, unfolded protein response (UPR), inositol-requiring enzyme 1 (IRE1)

## Abstract

Nitrogen (N) is of utmost importance for plant growth and development. Multiple studies have shown that N signaling is tightly coupled with carbon (C) levels, but the interplay between C/N metabolism and growth remains largely an enigma. Nonetheless, the protein kinases Sucrose Non-fermenting 1 (SNF1)-Related Kinase 1 (SnRK1) and Target Of Rapamycin (TOR), two ancient central metabolic regulators, are emerging as key integrators that link C/N status with growth. Despite their pivotal importance, the exact mechanisms behind the sensing of N status and its integration with C availability to drive metabolic decisions are largely unknown. Especially for SnRK1, it is not clear how this kinase responds to altered N levels. Therefore, we first monitored N-dependent SnRK1 kinase activity with an *in vivo* Separation of Phase-based Activity Reporter of Kinase (SPARK) sensor, revealing a contrasting N-dependency in *Arabidopsis thaliana* (Arabidopsis) shoot and root tissues. Next, using affinity purification (AP) and proximity labeling (PL) coupled to mass spectrometry (MS) experiments, we constructed a comprehensive SnRK1 and TOR interactome in Arabidopsis cell cultures during N-starved and N-repleted growth conditions. To broaden our understanding of the N-specificity of the TOR/SnRK1 signaling events, the resulting network was compared to corresponding C-related networks, identifying a large number of novel, N-specific interactors. Moreover, through integration of N-dependent transcriptome and phosphoproteome data, we were able to pinpoint additional N-dependent network components, highlighting for instance SnRK1 regulatory proteins that might function at the crosstalk of C/N signaling. Finally, confirmation of known and identification of novel SnRK1 interactors, such as Inositol-Requiring 1 (IRE1A) and the RAB GTPase RAB18, indicate that SnRK1, present at the ER, is involved in N signaling and autophagy induction.

## Highlights

• Interactome mapping under N starvation reveals SnRK1 signaling at the ER.
• SnRK1α1 phosphorylates the autophagy regulator RAB18 *in vitro*.
• SnRK1 interacts with the UPR regulator IRE1A, which is activated during N starvation.

## Graphical Abstract

**Figure:**
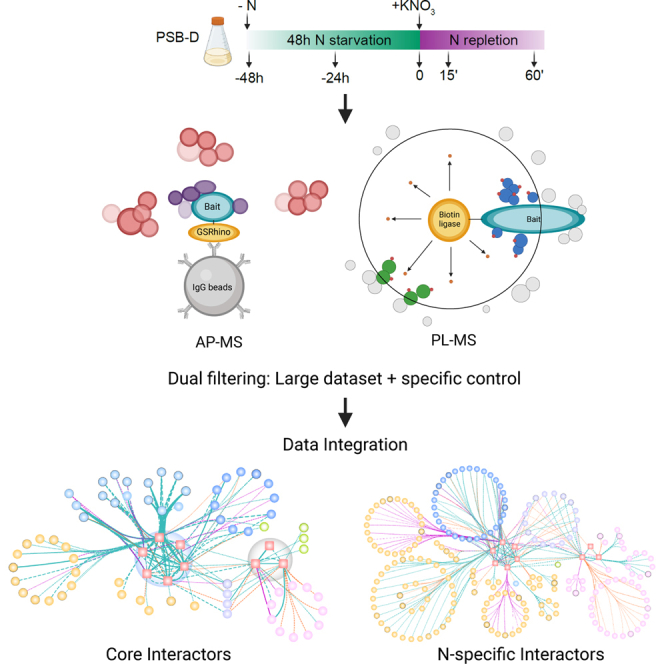

## In Brief

This manuscript presents the nitrogen-specific interactome of the plant SNF1-related Protein Kinase (SnRK1) and the Target of Rapamycin (TOR) kinase, utilizing Affinity Purification coupled to Mass Spectrometry (AP-MS) and Proximity Labeling coupled to Mass Spectrometry (PL-MS). Through data filtering and integration, the core and nitrogen-specific TOR and SnRK1 interactomes were delineated. Our study reveals the presence of nitrogen signaling by SnRK1 at the endoplasmic reticulum and demonstrates the nitrogen starvation-dependent activation of the novel SnRK1 interactor, Inositol-requiring enzyme 1A (IRE1A).

Nitrogen (N) is of utmost importance for plant growth and development, as it is an essential structural component of amino acids, the building blocks of proteins, and of nucleic acids. In addition, both inorganic N (NO_3_^-^, NH_4_^+^) as well as N-rich amino acids, such as glutamine, serve as signaling molecules that control plant developmental processes such as germination, seedling development, shoot growth, root architecture and flowering (1). Consequently, N levels in field conditions are one of the main limiting factors that determine plant yield (2). Because of its pivotal role, N uptake, assimilation and partitioning are tightly regulated in order to maintain metabolic homeostasis (3, 4). In plant metabolism, N levels are also coordinated with carbon (C) levels. As such, limiting N conditions result in reduced photosynthetic output, whereas increased C supply promotes N uptake and assimilation (5, 6). Disturbances in the C/N balance can also have detrimental effects for plant development, for instance, conditions of high C/low N inhibit post-germination growth of *Arabidopsis thaliana* (Arabidopsis) seedlings (7).

The TOR/SnRK1 signaling axis is one of the central pathways in primary metabolism that integrates N and C levels (8, 9). This regulatory hub consists of two kinase complexes that often work antagonistically to each other. When energy and nutrients are plentiful, the TOR kinase is active and growth and development are promoted, while during stress conditions, SnRK1 is activated, initiating energy conservation pathways for plant survival. These kinases are strongly conserved throughout all eukaryotic lineages, demonstrating their vital and central functions in cellular metabolism (10, 11). As autotrophs, however, plants consist of nutrient-providing source and nutrient-consuming sink tissues, necessitating a more complex interplay between both kinases to ensure dynamic regulation of nutrient partitioning. Therefore, the plant SnRK1 kinase has differentiated from its opisthokont orthologs, acquiring specific roles during plant development, next to its conserved function in stress responses (12, 13, 14).

TOR is a Ser/Thr protein kinase belonging to the family of phosphatidylinositol 3-kinase-related kinases. The plant TOR complex 1 (TORC1) is a heterotrimeric complex in Arabidopsis encoded by the catalytic TOR subunit and the regulatory subunits LETHAL WITH SEC THIRTEEN 8-1 (LST8-1) and REGULATORY-ASSOCIATED PROTEIN OF TOR 1 (RAPTOR1B) (15). So far, there is no clear evidence for the existence of a TORC2 *in planta*, in contrast to mammalian and yeast systems (15). The TOR kinase is active in nutrient-rich conditions, during which it promotes growth mainly through regulation of ribosome biogenesis and protein synthesis while suppressing energy-conserving processes such as autophagy (16). Because TOR is a major regulator of protein translation, it is strongly regulated by the availability of N and amino acids (17). In Arabidopsis, it was shown that the Rho-GTPase ROP2 promotes TOR activity in response to auxin (18) and N signals (19), and that NO_3_^-^, NH_4_^+,^ and amino acids each have the capacity to independently activate TOR after N depletion, however, the underlying molecular mechanisms remain elusive.

Downstream of TOR, it is not known if C and N have common or specific targets to regulate C/N-dependent plant growth. These processes are better understood in yeast, where a shift between N-poor and N-rich amino acid conditions induces specific TOR-dependent pathways to regulate the biosynthesis of amino acids and nucleotides, as well as C storage (20). Furthermore, in yeast, amino acids trigger spatially distinct pools of TOR in vacuoles and endosomes, initiating differential downstream processes (21). Similarly, in mammalian systems, amino acid sensing promotes fast subcellular translocation of the TOR kinase into lysosomal membranes (22). Intriguingly, homologs of the yeast and mammalian proteins involved in this translocation are unidentified in plants, and apart from the involvement of ROP2, it remains largely unknown how and where the plant TOR kinase senses internal N levels.

SnRK1 is the ortholog of the mammalian AMP-activated Protein Kinase (AMPK) and the yeast SNF1 kinase. In plants, the SnRK1 complex harbors a catalytic kinase subunit often indicated as the alpha subunit. In Arabidopsis, there are three genes encoding this catalytic subunit: SnRK1α1 (KIN10), SnRK1α2 (KIN11), and SnRK1α3 (KIN12). SnRK1α1 is the most broadly expressed subunit, followed by SnRK1α2, which can restore some functions in SnRK1α1 mutant lines, while SnRK1α3 is believed to be a pseudogene. Furthermore, the complex contains one of three regulatory beta subunits (SnRK1β1, SnRK1β2, and SnRK1β3). SnRK1β1 and SnRK1β2 contain N-terminal myristoylation sites, which can target SnRK1 complexes to membranes, while SnRK1β3 is an N-terminally truncated protein. Finally, the SnRK1 complex contains a plant-specific regulatory hybrid βγ subunit, encoded by the *SnRK1βγ* (*SNF4*) gene (13, 14). The catalytic activity of the α-subunit in the SnRK1 complex requires the phosphorylation of a specific threonine (T175 in Arabidopsis) residue in the highly conserved T-loop by the upstream SnRK1-activating kinases (SnAK1 and SnAK2) or *via* autophosphorylation (23). Moreover, it has been shown that SnRK1α1 can also be constitutively active and execute functions independent of its regulatory subunits (24).

Under C starvation and other stress conditions, SnRK1α1 can translocate from the endoplasmic reticulum (ER) to the nucleus to regulate downstream gene expression (24, 25), leading to massive transcriptional reprogramming (9) through phosphorylation of transcription factors such as bZIP63 (26). In yeast, N stress activates AMPK to reduce TOR activity, independently of AMP levels (27). In plants, however, it remains largely elusive how SnRK1 is influenced by N levels. One key substrate of SnRK1 is RAPTOR1B, and its phosphorylation by SnRK1 leads to the downregulation of TOR activity upon adverse conditions; however, a link with N deprivation was not analyzed (28). Downstream of SnRK1 and TOR signaling, nutrient starvation can be remediated through the induction of autophagy. Autophagy is a conserved degradation pathway needed for the recycling of damaged or unwanted cell materials upon stress conditions or during specific developmental processes (29, 30). As a negative autophagy regulator, TOR phosphorylates autophagy-related 13 (ATG13), part of the ATG1/ATG13 kinase complex that initiates phagophore formation. In contrast, under conditions of low C or N levels, SnRK1 activates autophagy by phosphorylation of ATG1 (31, 32). Moreover, SnRK1 can bypass TOR activity through phosphorylation of ATG6 (33), however, this pathway functions specifically during C starvation. Furthermore, a clade of specific FCS-like zinc finger (FLZ) proteins, which inhibit SnRK1 by repressing T-loop phosphorylation of the catalytic α-subunit (34), were shown to be transcriptionally repressed and actively targeted for autophagy-dependent degradation under energy depletion, hereby constituting a positive feedback mechanism between SnRK1 and autophagy (35). The presence of both TOR-dependent as well as -independent regulation of autophagy demonstrates the tight regulation of this process in relation to the C/N balance (31).

While a lot of data are available about how C signals influence SnRK1 localization and activity (9, 24, 26, 36), this information is much more limited for N. Nonetheless, it has been shown that SnRK1α1 phosphorylates NIN-like protein 7 (NLP7), a master regulator of the primary nitrate response (PNR) (37), promoting its cytoplasmic localization and subsequent degradation under N-limiting conditions (38). Another SnRK1 substrate that is involved in the regulation of the C/N balance is the transcription factor bZIP63 (39), which activates transcription of the gene encoding the electron-transfer flavoprotein:ubiquinone oxidoreductase, leading to altered branched chain amino acid catabolism and thereby regulation of C and N pools (26). SnRK1 has also been implicated in the regulation of flowering in response to N levels through phosphorylation of the transcription factor FLOWERING BHLH 4 (40). Furthermore, there is evidence that SnRK1 is associated with primary C/N signaling *via* the transcriptional regulation of C/N-regulatory kinases (5). Finally, the SnRK1β1 subunit displays transcriptional regulation by ammonium nitrate, hinting towards a specific role of this subunit in N signaling (41).

Recent studies have mapped the TOR and SnRK1 phosphoproteome and interactome in the context of sucrose signaling (36, 42). In these studies, a comprehensive TOR-dependent phosphoproteome was mapped under differential sucrose conditions, and AP-MS was used to identify TOR complex interactors (42). For SnRK1, rapid dephosphorylation upon sucrose re-supply to sucrose-starved cells enabled the identification of C-dependent SnRK1 targets. Moreover, a combination of AP-MS and PL-MS was employed to shed light on the C-related SnRK1 interactome (36). As these studies were focused mainly on C signaling, only a few links with N signaling were uncovered, such as the identification of NLP1 and the nitrate reductase NIA2 as SnRK1 substrates (36).

In summary, research in plants on how N metabolism is integrated with these central SnRK1/TOR signaling pathways is lagging in the mammalian and yeast fields (43, 44). To close this knowledge gap, we first investigated the N-dependency of cytosolic SnRK1 activity in different plant tissues using our recently developed *in vivo* SPARK-based SnRK1 kinase reporter (45), revealing contrasting activities in shoots and roots that are most likely linked with source/sink dynamics. Then, after establishing that also SnRK1 responds to N levels, the N-dependent networks around SnRK1 and TOR were mapped through interactomics, using Arabidopsis cell suspension cultures as a homogenous system for in-depth network mapping. Therefore, an N-starvation/KNO_3_-repletion assay for Arabidopsis cell cultures was developed. Monitoring of TOR and SnRK1 activities and N-responsive marker genes clearly showed the conservation of N-dependent responses in this system. Using this assay, the SnRK1/TOR interactome was mapped under N-starved and KNO_3_-repleted conditions using a combination of AP-MS and PL-MS. This network was thoroughly compared to the corresponding C-dependent networks, discovering N-specific interaction partners of these central signaling modules. Furthermore, data integration with publicly available N-dependent transcriptome and phosphoproteome datasets pinpointed interactors that might be linked with N signaling and/or with the integration of the C/N balance. Identification of some novel signaling components suggests that SnRK1-dependent N signaling occurs at the ER, and points toward autophagy induction as one of the main N-dependent downstream target processes.

## Experimental Procedures

### Experimental Design and Statistical Rationale

For the SPARK assays, quantification was performed on five seedlings per condition and per timepoint (n = 5), or three biological replicates in PSB-D cell culture (n = 3). RT-qPCR results obtained in PSB-D cell culture were derived from three biological replicates (n = 3) and normalized against two reference genes. For analysis of S6K phosphorylation in PSB-D cell culture, representative immunoblots are shown of three independent biological replicates (n = 3). For all AP-MS and TurboID-MS analyses, samples were derived from transgenic Arabidopsis PSB-D cell suspension cultures. Each transgenic cell culture consists of a mixture of numerous independent transformation events, leading to highly reproducible transgene levels, as was shown before (46). Consequently, limited biological variation can be expected in this homogenous system, thus, to evaluate reproducibility and quantitative accuracy, three technical replicates (n = 3) were conducted and subjected to statistical analysis using the reported methods. Radioactive *in vitro* kinase assays were performed in triplicate (n = 3), whereas *in vitro* kinase assays followed by MS were conducted in duplicate (n = 2). For all kinase assays, corresponding negative controls with inactive SnRK1α1 kinase (K48M mutant) was included. The SPPIER confocal images show representative results of at least three independent infiltrations (n = 3). For the IRE1 activity assay, representative RT-PCR results are shown of three independent biological replicates (n = 3), and Actin transcripts were monitored as a reference.

### Separation of Phase-Based Activity Reporter of Kinase (SPARK) Assay

Transgenic Arabidopsis Col-0 plants and the PSB-D cell culture expressing the AMPK substrate peptide (ASP)-SPARK construct were previously described (45). ASP-SPARK plants were grown on ½ Murashige & Skoog (MS) medium without sucrose. After 5 days of growth under long-day conditions (16 h light at 21 °C, 8 h dark at 18 °C), seedlings were transferred to either fresh ½ MS medium (high N, 1900 mg/L KNO_3_, 1650 mg/L NH_4_NO_3_) or ½ MS medium without N (0 mM KNO3, 0 mM NH4NO;, M531 PhytoTech Labs). Plants were imaged by an Olympus FluoView1000 (FV1000 ASW) confocal laser scanning microscope using a Plan-Apochromat 20 × /0.75 dry objective lens.

PSB-D cell cultures were grown and maintained as described in (47). For the N-starvation experiments, PSB-D cells were washed three times with N-free medium (0 mM KNO_3_, 0 mM NH_4_NO_3_, MSP07 Caisson) containing 0.5% (w/v) sucrose on a glass filter funnel without drying of cells and resuspended in N-free medium. After 48 h, 1 ml of N-starved culture was added to 1 ml of liquid full MSMO medium (18,79 mM KNO_3_, 20,61 mM NH_4_NO_3_, Duchefa) or to 1 ml N-free medium as control, with 1% (w/v) low melting point agar and poured into a Nunc Lab-Tek II chambered cover glass. Immediately after solidification, the imaging chamber was mounted and imaging of three randomly selected cell clumps was started at a rate of one image per 90 s on a Zeiss LSM710 Zeiss microscope equipped with a Plan-Apochromat 20×/0.8 dry objective.

In all SPARK experiments, eGFP was excited with an Argon laser at 488 nm and emission was acquired in the range from 500 to 530 nm. mCherry was excited with a solid-state 559-nm laser and emission light was acquired between 600 and 630 nm. The pinhole was set to 1 Airy unit, and the images were acquired at 512 × 512 pixels resolution using line sequential scanning. The SPARK signal was quantified using the FIJI software as described (45); graphs were made using GraphPad Prism.

### Cloning and Transformation

All oligonucleotides used for cloning are listed in Supplemental Table S1*A*. All open reading frames were cloned into GreenGate (48) pGGC000 entry vector using NEBuilder HiFi DNA Assembly. The resulting entry clones were sequence-verified and used as a template for site-directed mutagenesis to remove internal *Bsa*I sites if present. For interactomics, all GS^Rhino^ and linkerTurboID-3xHA tag fusion constructs were either obtained from (36, 42) or *Bsa*I Golden Gate cut-ligation was used to make N- or C-terminal fusions under control of the 35S promotor and 35S terminator, as described previously (36). Expression vectors were transferred to the *Agrobacterium tumefaciens* strain C58C1 RifR pMP90 for transformation into the Arabidopsis PSB-D cell culture (49). The coding sequences of BSK4, IRE1A_cyt, NLP8, PANK2, RAB18, and SEC12 were cloned into pGGC000, and GreenGate cloning was used to make N-terminal 6xHis-MBP (Maltose Binding Protein) or C-terminal MBP-6xHis (for BSK4) fusions, or separation of phase-based protein interaction reporters (SPPIER) HOTag fusions (45).

### The N-Starvation/KNO_3_-Repletion Assay in the PSB-D Cell Culture

Dark-grown PSB-D Arabidopsis cell cultures (wild type (WT) or 35S: S6K-3xHA) were grown and maintained as described in (49), weekly sub-culturing cell cultures in fresh MSMO medium. For N starvation, 3-day old cell cultures were washed three times with MSMO without N (Caisson labs, MSP07) containing 0.5% (w/v) sucrose (N-free medium) by vacuum filtration on a glass filter funnel without allowing the cells to dry. Cells were diluted two-fold in N-free medium, grown in the absence of N for 48 h, and then the N source was added and the culture was further grown for 2h.

For reverse transcription quantitative PCR (RT-qPCR) analysis of SnRK1, N starvation, and PNR marker genes, WT PSB-D cells were N-starved for 48 h. Samples were taken at 0 h, 24 h, and 48 h after washing away the N, and 15 min, 45 min, and 120 min after KNO_3_ addition. RNA was extracted using the ReliaPrep RNA Miniprep System (Promega) and cDNA was prepared with the qScript cDNA Synthesis Kit (QuantaBio), according to the supplier’s instructions. Primers used for RT-qPCR can be found in Supplemental Table S1*B*. RT-qPCR was performed on the LightCycler 480 Real-Time SYBR Green PCR System (Roche) and expression was analyzed through the 2^-ΔΔCT^ method. Two reference genes were used for normalization (*ACT2*, AT3G18780; *EF1ALPHA*, and AT5G60390). Graphs with relative expression levels were made using GraphPad Prism.

For analysis of TOR activity, the 35S: S6K1-3xHA culture was obtained from (42). Samples were taken 15 min, 30 min, 45 min, 1 h, and 2 h after the re-addition of an N source (1 mM KNO_3_, 1 mM NH_4_Cl, or 1 mM Gln) to 48 h N-starved PSB-D cells; 1 mM KCl was used as mock negative control. In the AZD8055 negative control samples, 1 μM of the TOR inhibitor AZD8055 was added 1 h before addition of the corresponding N source. Proteins were extracted as described previously (42), total protein extracts were separated by SDS-PAGE and proteins were transferred by immunoblotting. Immunoblots were blocked overnight in 3% (w/v) skimmed milk in TBS-T buffer and detected with mouse anti-HA (1/2000; 1 h) (clone 12CA5, Roche) as the primary antibody and anti-mouse-HRP (1/10,000; 1 h) (NA931, GE Healthcare) as the secondary antibody.

### Arabidopsis Cell Culture Transformation and Biomass Generation for Interactomics

All GS^rhino^ and linkerTurboID-3xHA fusion constructs were transferred to *A. tumefaciens* C58C1 RifR (pMP90) and transformed into the dark-growing Arabidopsis PSB-D cell culture. Expression of bait fusion proteins was checked by total protein extraction followed by SDS-PAGE and immunoblotting with Peroxidase Anti-Peroxidase Soluble Complex antibody (1/2500; 1h) (P1291, Sigma) for AP-MS GS^rhino^ fusions or rabbit Anti-BirA (1/5000; overnight) (mutated/TurboID) (AS20 4440, Agrisera) and anti-rabbit-HRP (1/10,000; 1h) (NA934, Cytiva) for PL-MS TurboID-3xHA fusions (Supplemental Fig. S1). Next, cultures were maintained by weekly sub-culturing into fresh MSMO medium and further upscaled as described (47, 49). Three days after sub-culturing into nutrient-rich MSMO medium, cells were washed and resuspended in N-free medium containing 0.5% (w/v) sucrose. After 48 h of N starvation, cultures were split into two shake flasks, and 1 mM KNO_3_ was added to one while the other served as N-starvation control. For the TurboID cultures, 50 μM biotin was added 48 h after N starvation, and cells were grown further for 15 min or 1 h with or without KNO_3_ addition. Cells were harvested through vacuum infiltration, sampling 7.5 g of PSB-D cells for triplicate AP-MS experiments and 9 g PSB-D cells for triplicate PL-MS experiments. Harvested cell material was frozen in N_2_ and stored at −70 °C.

### Isolation of Protein Complexes by GS^rhino^-Based Affinity Purification

For AP-MS, total protein extracts were prepared in extraction buffer (25 mM Tris–HCl (pH 7.6), 15 mM MgCl_2_, 150 mM NaCl, 15 mM p-nitrophenyl phosphate, 60 mM β-glycerophosphate, 0.1% (v/v) NP-40, 0.1 mM Na_3_VO_4_, 1 mM NaF, 1 mM PMSF, 1 μM E64, EDTA-free Ultra Complete tablet (Roche) and 5% (v/v) ethylene glycol), starting from 2.5 g frozen cell material per replicate, as described previously (42). GS^rhino^-based affinity purification experiments were performed in triplicate, as reported before (36, 49). Briefly, protein complexes were trapped through the Protein G part of the GS^rhino^ tag by incubating 25 mg total protein extract per replicate for 45 min with 50 μl in-house prepared magnetic immunoglobulin G (IgG) bead suspension. Beads were washed three times with 500 μl extraction buffer, once with 500 μl extraction buffer without detergent, and once with 800 μl 50 mM NH_4_HCO_3_ (pH 8.0). The wash buffer was removed and beads were incubated in 50 μl 50 mM NH_4_HCO_3_ supplemented with 1 μg Trypsin/Lys-C (Promega) for 4 h at 37 °C. Next, the digest was separated from the beads and further incubated overnight with 0.5 μg Trypsin/Lys-C at 37 °C. Finally, the digest was centrifuged at 20,800 rcf for 5 min, and supernatant was dried in a SpeedVac and stored at −20 °C until MS analysis.

### TurboID-Based Proximity Labeling

Proximity labeling experiments were performed in triplicate, as previously described in detail for Arabidopsis PSB-D cells (50) (36). Briefly, proteins were extracted starting per replicate from 3 g harvested cell material using denaturing extraction buffer (100 mM Tris-HCl (pH 7.5), 2% (w/v) SDS, 8 M Urea). After removal of free biotin, biotinylated proteins were isolated by incubating the entirely derived protein extract with Streptavidin Sepharose beads. Finally, proteins were on-bead digested with Trypsin/Lys-C, and biotinylated peptides were further eluted through acid elution.

### LC−MS/MS Analysis

On-bead digested samples were analyzed on a Q Exactive (ThermoFisher Scientific) using standard procedures (see (36, 42, 47, 49)). Briefly, peptides were redissolved in loading solvent A (0.1% TFA in water/ACN (98:2, v/v)) and injected for LC-MS/MS analysis on an Ultimate 3000 RSLC nano LC (Thermo Fisher Scientific) in-line connected to a Q Exactive mass spectrometer. The peptides were first loaded on a trapping column (made in-house, 100-μm internal diameter (I.D.) × 20 mm, 5-μm beads C18 Reprosil-HD, Dr Maisch, Ammerbuch-Entringen, Germany), and after flushing from the trapping column, the peptides were separated on a 50-cm μPAC column with C18-endcapped functionality (Pharmafluidics, Belgium) kept at a constant temperature of 50 °C. Peptides were eluted by a nonlinear gradient from 99% solvent A’ (0.1% formic acid in water) to 10% solvent B′ (0.1% formic acid in water/ACN, 20/80 (v/v)) in 9 min, 30% solvent B′ in 29 min, 50% solvent B′ in 34 min at a flow rate of 300 nl/min, followed by a 5-min wash reaching 95% solvent B’. The mass spectrometer was operated in data-dependent, positive ionization mode, automatically switching between MS and MS/MS acquisition for the five most abundant peaks in a given MS spectrum.

### Data Analysis and Background Filtering

From Thermo raw data files, Mascot Generic Files were created using the Mascot Distiller software (versions 2.5.0.0 and 2.8.2.0, Matrix Science). When generating these peak lists, grouping of spectra was allowed with a maximum intermediate retention time of 30 s, and a maximum intermediate scan count of five was used where possible. Grouping was done with 0.005 Da precursor tolerance. A peak list was only generated when the MS/MS spectrum contained more than 10 peaks. There was no de-isotoping and the relative signal-to-noise limit was set to 2. Homology-based protein identification was done using the Mascot search engine (versions 2.6.2 and 2.8.2, Matrix Science) with database Araport11plus. This database contains all entries from the Araport11 database (The Arabidopsis Information Resource, www.arabidopsis.org/) extended with sequences of different types of possible contaminants in proteomics experiments. These include the common Repository of Adventitious Proteins (cRAP) protein sequences, a list of proteins commonly found in proteomics experiments (The Global Proteome Machine, www.thegpm.org/crap/). Additionally, commonly used tag sequences and typical purification contaminants, such as sequences derived from the resins or the used proteases, were added. The Araport11plus database contains in total 49,055 sequence entries. Variable modifications were set to methionine oxidation, acetylation of protein N-termini, and for PL experiments, also biotinylation of lysine. Mass tolerance on MS was set to 10 ppm (with Mascot’s C13 option set to 1) and the MS/MS tolerance at 20 mmu. The peptide charge was set to 2+, 3+ and 4+ and the instrument setting was set to ESI-QUAD. Trypsin was set as the protease used, allowing for two missed cleavages, and cleavage was also allowed when arginine or lysine is followed by proline. Only high-confident peptides, ranked 1 and with scores above the threshold score, set at 99% confidence, were withheld. Protein identifications were initially retained for further filtering, if they were identified by at least one unique peptide with a PSM confidence of >99.

The Thermo raw files were also processed using the MaxQuant software (versions 1.6.10.43 and 2.2.0.0) (51). Data were searched with the built-in Andromeda search engine against the Araport11plus database. For AP-MS, variable modifications were set to methionine oxidation, acetylation of protein N-termini, and phosphorylation of serine, threonine, and tyrosine. For PL-MS, variable modifications were set to methionine oxidation, acetylation of protein N-termini, phosphorylation of serine and threonine, and biotinylation of lysine, and dependent peptide search was on. Mass tolerance on precursor ions was set to 4.5 ppm and on fragment ions to 20 ppm. The enzyme was set to trypsin/P, allowing for two missed cleavages, and cleavage was allowed when arginine or lysine was followed by proline. PSM and protein identifications were filtered using a target-decoy approach at a false discovery rate (FDR) of 1%. Proteins identified with at least one unique peptide were retained.

For the identification of bait-specific interactors in the AP-MS data, we took advantage of a large in-house control AP-MS dataset derived from Mascot analyses with 515 AP-MS cell culture experiments on 109 bait proteins, not related to SnRK1 or TOR signaling and grouped functionally into 28 bait-groups. This large control dataset includes triplicate negative control AP-MS experiments on PSB-D WT cell cultures, harvested either under N-starvation or KNO_3_-repletion conditions. As a first filter, the abundance of each identified protein was compared against its average abundance in the control dataset. Hereto, normalized spectral abundance factors (NSAF) were calculated for all identified proteins and averaged over the three technical repeats. In parallel, the corresponding average NSAF of each protein was calculated from the control dataset, after imputation of missing values by a constant value of 0.001. Next, the ratio of the average bait and control NSAF values was determined as a measure of fold enrichment, and a two-tailed Student’s *t* test was performed comparing bait and control Ln-transformed NSAF values to determine the enrichment significance. *t* test *p*-values were -Log_10_ transformed and infinite values were replaced by a constant -Log_10_ *p*-value of 325. Identifications were considered significantly enriched if they passed one of the following criteria: (i) two-peptide identifications present in at least two out of three replicates are significantly enriched if they were found with less than three other bait-groups in the large control AP-MS dataset or if they were enriched with a mean NSAF ratio ≥10 AND a -Log_10_(*p*-value) ≥ 10, or with a mean NSAF ratio ≥20 AND a -Log_10_(*p*-value) ≥ 8, (ii) one-peptide identifications present in all three replicates, if they were found in the large control dataset with less than five bait-groups and if they were significantly enriched with a mean NSAF ratio ≥10 AND a -Log_10_(*p*-value) ≥ 10. As a second, additional filter, average NSAF values were compared to the average NSAF value over the six WT PSB-D negative control experiments. Hereto, the triplicate N-starved and triplicate KNO_3_-repleted experiments were grouped as one negative control dataset. In this filtering, proteins were retained as specific if their Affinity Enrichment Score (AES), that is, the product of their NSAF ratio and their -Log_10_(*p*-value), was ≥100.

For the identification of specific proteins in the PL-MS data, we used a similar approach integrating a large in-house control PL-MS dataset derived from Mascot analyses on 283 PL-MS cell culture experiments covering 37 bait proteins not related to SnRK1 or TOR signaling. This control dataset includes triplicate negative control PL-MS experiments on PSB-D WT and 35S: GFP-TurboID cell cultures harvested under similar N-starvation/KNO_3_-repletion conditions as the bait proteins and treated with 50 μM biotin for 15 min (WT) or 1 h (GFP-TurboID). For the first filter, NSAF ratios and -Log_10_(*p*-values) were calculated for all identified proteins as described for the AP-MS analysis. Missing values in the control dataset were replaced by a constant NSAF value of 0.0005. Next, Affinity Enrichment Scores were calculated and proteins with an AES ≥20 were retained. As an additional control to correct for proteome changes provoked by the N-starvation/KNO3-repletion condition, a second filtering was implemented. Hereto, all bait experiments were analyzed by MaxQuant Label-Free Quantification (LFQ) analysis, together with their corresponding negative controls (WT or GFP-TurboID) harvested under similar conditions. The MaxQuant proteingroup.txt files were uploaded into Perseus (version 1.6.15.0) (52) and analyzed as described before (36, 42). Triplicate bait and negative control experiments were separately grouped and compared through Volcano plot analysis (FDR = 0.01, S0 = 1) to detect specific interactors for each PL-MS experiment (Supplemental Fig. S3*B*). Finally, this MaxQuant LFQ analysis was integrated with the NSAF dataset of the first filtering strategy, retaining only filtered proteins from the NSAF dataset that were at least once significantly differing in the MaxQuant LFQ analysis with a bait protein of the same complex (SnRK1 or TOR). Proteins identified by only one unique peptide were retained if the protein was identified in all three repeats, or if the protein was identified with one unique peptide in at least two replicates and additionally found with at least two peptides in another PL-MS experiment with a bait protein of the same complex.

For LFQ-based differential analysis between N-starved and KNO3-repleted conditions in AP-MS or PL-MS, the MaxQuant proteingroup.txt files were used. Thereto, for each bait, N-starved and KNO3-repleted LFQ intensity columns, all in triplicate, were uploaded into Perseus (version 1.6.15.0), reverse, only identified by site and contaminants already removed. LFQ intensities were log2 transformed, N-starved and KNO3-repleted experiments were separately grouped, and identifications were filtered for a minimal 3 valid values in at least 1 group. Missing values were imputed with values drawn from a normal distribution, using a width of 0.3 and a downshift of 1.8. For each bait, the N-starved condition was compared with the KNO3-repleted condition through Volcano plot analysis, using thresholds FDR = 0.05, S0 = 1.

### SnRK1 Consensus Motif Analysis

For proteome-wide *in silico* prediction of SnRK1 consensus motifs, Arabidopsis protein sequences (TAIR10 annotation) where queried for the presence of one of the two known SnRK1 consensus phosphorylation motifs (53) ([FILMV]X[RKH]XX[ST]XXX[FILMV] or [FILMV]RXXX[ST]XXX[FILMV]) through the Patmatch tool available at The Arabidopsis Information Resource. The significance of enrichment among the SnRK1 and TOR networks was determined through hypergeometric distribution analysis.

### GO Enrichment Analysis

ShinyGO (version 0.80) was used for the GO enrichment analysis (54). The novel and/or N-specific SnRK1/TOR subnetwork (without core subunits) was entered as input, and enriched biological processes were analyzed with an FDR cut-off of 0.05, against Araport11 protein-encoding genes as background. Finally, a selection of the output from the GO term biological process was presented using GraphPad Prism.

### Network Visualization

All interactome and co-expression networks were visualized in Cytoscape (version 3.10.0), using node and edge attributes as listed inSupplemental Table S3, *C* and *D*. For comparison of the N- and C-related interactome data (Supplemental Figs. S7 and S8), dot plots representing absolute and relative NSAF values were generated using ProHits-viz (46).

### Co-expression Analysis

Co-expression analysis was done on the genes of the corresponding proteins using Cornet (version 3.0) (55). Genes were considered co-expressed if the PCC ≥0.75 in at least two transcriptome compendia. The network was visualized using Cytoscape as described earlier, integrating the PCC values and the number of transcriptome compendia in the color and width of the edges, respectively.

### Kinase Assays

For recombinant protein production, His-MBP expression vectors were transformed into *Escherichia coli* strain BL21. A preculture of transfected cells was grown overnight at 37 °C in 4 ml Luria Broth (LB) medium (Invitrogen). The next day, the preculture was added to 250 ml LB medium and grown further at 37 °C until an OD_600_ between 0.4 and 0.6 was reached. Bacterial cultures were cooled to room temperature before the addition of 0.4 mM isopropyl-β-D-thiogalactoside. Cells were incubated overnight at 18 °C for induction of recombinant proteins. Growth medium was removed by centrifugation and cell pellets were stored at −70 °C. Cell pellets were incubated for 1 h at 4 °C in 17 ml lysis buffer (1×PBS buffer, 1 mM dithiothreitol, 1 mM EDTA, 1×protease inhibitor cocktail (Roche), 1% (v/v) Triton X-100 and 1 mg/ml Lysozyme). The cell lysate was sonicated on ice for 5 min (10 s ON, 10 s OFF), centrifuged twice at 17,096 rcf for 15 min, and the resulting supernatant was filtered with a prefilter and a 0.40-μm syringe filter (Minisart). For MBP-based purifications, the filtered protein extract was incubated for 1 h with 200 μl amylose resin (NEB) slurry. Beads were washed three times with 10 ml wash buffer (1×PBS, 1 mM EDTA and 1 mM dithiothreitol). For elution, beads were incubated three times for 5 min with 500 μl elution buffer (1×PBS, 1 mM dithiothreitol, 0.1% (v/v) Triton X-100 and 15 mM maltose). Proteins were concentrated with an Amicon Ultra centrifugal filter unit (MW cut-off 30 kDa; Millipore) until a volume of 250 μl was reached. Glycerol was added to a final concentration of 15% (v/v) and proteins were stored at −70 °C.

For *in vitro* SnRK1 kinase assays, recombinant substrates were incubated with His-MBP-SnRK1α1 or His-MBP-SnRK1α1-K48M (kinase-dead). Kinase reactions were performed for 1 h at 30 °C, combining 1 to 4 μl kinase with 2 to 6 μl substrate in kinase assay buffer (50 mM Tris-HCl (pH 8.0), 1 mM EGTA, 2.5 mM DTT, 5 mM MgCl_2_, 50 μM cold ATP, 1x PhosSTOP), supplemented with 5 μCi γ-^32^P ATP. Reactions were stopped by addition of SDS sample buffer and incubated for 10 min at 95 °C. Proteins were separated by SDS-PAGE and stained with Coomassie brilliant blue R-250. Gels were dried and radioactivity was detected by autoradiography on a photographic film.

For kinase assays coupled to mass spectrometry, recombinant substrate fusion proteins were combined with His-MBP-SnRK1α1 or His-MBP- SnRK1α1-K48M (kinase-dead) as described earlier, but now incubated overnight at 30 °C in kinase assay buffer without γ-^32^P ATP. Reactions were stopped by the addition of NuPAGE LDS sample buffer (NP0007, Invitrogen) and NuPAGE Sample reducing agent (MP0004, Invitrogen) and incubated for 10 min at 70 °C. Proteins were separated by SDS-PAGE on a NuPAGE gel and stained with Coomassie Brilliant Blue G-250. Gel excision and preparation of the samples for MS was done as described in (47).

For LC−MS/MS analysis, peptides were re-dissolved in 20 μl loading solvent A (0.1% TFA in water/ACN (98:2, v/v)), of which 4 μl was injected for LC-MS/MS analysis on an Ultimate 3000 RSLC nano LC (Thermo Fisher Scientific) in-line connected to a Q Exactive mass spectrometer (Thermo Fisher Scientific). The peptides were first loaded on a trapping column that was made in-house, 100 μm internal diameter (I.D.) × 20 mm, 5 μm beads C18 Reprosil-HD (Dr Maisch). After flushing from the trapping column, peptides were separated on a 50 cm μPAC column with C18-endcapped functionality (Pharmafluidics) kept at a constant temperature of 50 °C. Peptides were eluted by a non-linear gradient from 99% solvent A’ (0.1% formic acid in water) reaching 10% solvent B’ (0.1% formic acid in water/ACN, 20/80 (v/v)) in 9 min, 33% solvent B′ in 32 min, 55% solvent B′ in 43 min, 70% solvent B′ in 48 min, at a flow rate of 300 nl/min, followed by a 2 min wash with 70% solvent B’. The mass spectrometer was operated in data-dependent, positive ionization mode, automatically switching between MS and MS/MS acquisition for the 5 most abundant peaks in each MS spectrum. Full-scan MS spectra (400–2000 m/z) were acquired at a resolution of 70,000 in the Orbitrap analyzer after accumulation to a target value of 3,000,000. The 5 most intense ions above a threshold value of 13,000 were isolated with a width of 2 m/z for fragmentation at a normalized collision energy of 25% after filling the trap at a target value of 50,000 for maximum 80 ms. MS/MS spectra (200–2000 m/z) were acquired at a resolution of 17,500 in the Orbitrap analyzer.

The Thermo raw files were searched with MaxQuant (version 2.2.0.0) against the Araport11plus database with recombinant His-MBP fusion protein (substrates and kinases) sequences added. Variable modifications were set to oxidation(M), acetyl (protein N-termini), and phospho(S,T,Y). Mass tolerance on precursor ions was set to 4.5 ppm and on fragment ions to 20 ppm. Trypsin was set as protease, allowing for 2 missed cleavages, and cleavage when R or K was followed by P. PSM and protein identifications were filtered using a target-decoy approach at a false discovery rate (FDR) of 1%.

### SPPIER

The SnRK1α1-mCherry-HOTag3 construct and the Greengate entry plasmids containing eGFP-HOTag6 were described before (45). The coding sequences of IRE1A_cyt, NLP8 and BSK4 were cloned into pGGC000, and GreenGate cloning was used to make the N- and C-terminal eGFP-HoTag6 fusions under the control of the 35S promotor and terminator. *Nicotiana benthamiana* plants were grown for 4 weeks in the greenhouse. Leaves were infiltrated with *A. tumefaciens* C58C1 RifR (pMP90) cells at an OD_600_ of 0.5 for each construct, dissolved in infiltration buffer (100 μM acetosyringone, 10 mM MgCl_2_, 10 mM MES (pH5.7)), using a 1-mL syringe. A p19 antiviral suppressor gene was co-infiltrated to suppress viral defence responses (56). Two to 3 days after transformation, leaf excisions were imaged *via* confocal microscopy (Zeiss LSM710 or Olympus FluoView1000), as described above in the SPARK experimental procedures.

### IRE1 Activity Assay

Plants were grown on ½ Murashige & Skoog (MS) medium without sucrose. After 5 days of growth under long-day conditions (16 h light at 21 °C, 8 h dark at 18 °C), seedlings were transferred to either fresh ½ MS medium (high N) or ½ MS medium without N (M531 PhytoTech Labs), identical to the SPARK assay. Plants were pooled and collected, RNA was extracted using the ReliaPrep RNA Miniprep System (Promega) and cDNA was prepared with the qScript cDNA Synthesis Kit (QuantaBio), according to the supplier’s instructions. Primers used for RT-PCR of the bZIP60 splice variant and of BiP3 can be found in Supplemental Table S1*C*. RT-PCR was performed at an annealing temperature of 55 °C for 40 cycles and samples were run on an 1.2% (w/v) agarose gel at 100 V for 30 min.

## Results

### SnRK1 Activity Is Regulated by N in a Tissue-specific Manner

**Fig. 1:**
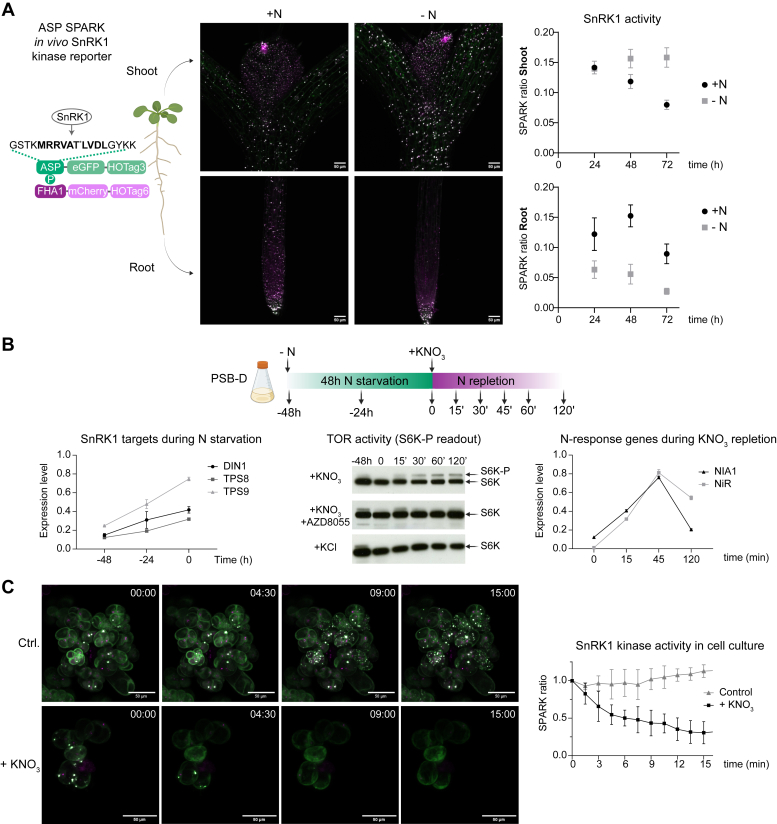
**Exploring N-dependent dynamics in SnRK1 and TOR activity in Arabidopsis.***A*, the ASP-SPARK reporter shows a dynamic SnRK1 activity in response to altered N levels in the shoot and root of seedlings. Five-day-old seedlings were transferred to ½ MS with (+) or without (−) N and imaged 72 h after transfer. Scale bars, 50 μm. The graphs show the quantification of the SPARK ratio in root and shoot 24 h, 48 h and 72 h after transfer (mean ± SEM, n = 5). *B*, evaluation of KNO_3_-dependent TOR and SnRK1 activity in PSB-D cell cultures. The *top panel* illustrates the N-starvation/KNO_3_-repletion strategy, together with the timepoints at which samples were taken. The timepoint −48 h corresponds to cells 3 days after sub-culturing into fresh MSMO medium, just prior of the start of N starvation, whereas timepoint 0 represents the sample taken just before addition of KNO_3_. The *left* and *right* graph show normalized expression levels of SnRK1 target genes during N starvation or N-responsive genes during KNO_3_ repletion, respectively (mean ± SEM, n = 3). The middle panel shows anti-HA immunoblot results of the KNO_3_- and TOR-dependent S6K1-3xHA phosphorylation (S6K-P). As negative controls, cultures were pretreated for 1 h with the TOR-inhibitor AZD8055 before KNO_3_ or KCl was applied. *C*, time-lapse confocal imaging of the ASP-SPARK sensor in N-starved PSB-D cell cultures after KNO_3_ resupply. The control (Ctrl.) cell culture did not receive KNO_3_. Scale bars, 50 μm. The graph at the *right* shows the quantification of the SPARK ratio for the non-treated control and after re-addition of 1 mM KNO_3_ (mean ± SEM, n = 3).

Prior to mapping N-dependent TOR and SnRK1 interactions, we first investigated the poorly understood association of SnRK1 with N signaling in plants in more detail. Using the recently developed SPARK-based sensor of SnRK1 activity (45), we monitored the N-dependent dynamics of SnRK1 *in planta* at an unprecedented resolution. This reporter relies on the specific phosphorylation of a synthetic AMPK substrate peptide (ASP) by SnRK1 and exploits phosphorylation-induced phase separation of fluorescent SPARK droplets as a proxy for SnRK1 kinase activity in the cytosol (45). To visualize tissue-specific responses of SnRK1 to varying N levels, Arabidopsis seedlings were grown for 5 days on ½ MS, after which they were transferred to medium with or without N. Next, seedlings were imaged by confocal microscopy 24, 48 and 72 h after transfer and the SPARK signal was quantified (Fig. 1*A* and Supplemental Fig. S2*A*).

In 5-day-old seedlings, SnRK1 still has a high basal activity in both shoots and roots, in agreement with its role in seedling establishment (57). Later on during development, when the seedlings become autotrophic and when sufficient N is available to support growth, SnRK1 activity in the shoot gradually decreases, with some constitutive activity remaining mainly in the zone near the shoot apical meristem. Upon N starvation, and in contrast to the control (stable N), SnRK1 activity remained high in the whole shoot until 72 h after N removal (Fig. 1*A* and Supplemental Fig. S2*A*), which is consistent with the expected growth arrest under N deficiency (19). In the roots, however, the N-dependent SnRK1 responses were different. While SnRK1 activity in the root apical meristem remained high regardless of N availability in the medium, SnRK1 activity in the root elongation zone exhibited a contrasting pattern to that observed in the shoots, showing a gradual decrease during N starvation (Fig. 1*A* and Supplemental Fig. S2*A*). This attenuation of SnRK1 activity might be linked with an increase in TOR activity (58, 59) to promote primary root growth and elongation during the N foraging response when soil nutrients such as N or phosphate become scarce.

### SnRK1 and TOR Respond to Altered N Levels in Arabidopsis Cell Culture

Previously, we have successfully mapped a sucrose-dependent signaling network around the TOR and SnRK1 kinases using sucrose-synchronized Arabidopsis PSB-D cell suspension cultures (36, 42). Based on these results, and taking the known N-dependent regulation of both TOR (19) and SnRK1 (see above) into account, we decided to use this homogeneous system to explore the protein interactome around both kinases in response to varying N conditions (6, 7). To assess how TOR and SnRK1 respond to altered N levels in this system, we first developed an N-starvation/KNO_3_-repletion assay to monitor their activities (Fig. 1*B*).

As a direct readout for TOR activity, phosphorylation of the canonical TOR substrate ribosomal S6 Kinase (S6K) was determined by detecting a phosphorylation-induced band shift through immunoblotting, whereas SnRK1 responses were measured by RT-qPCR analysis of known SnRK1 target genes. Based on these readouts, conditions of maximal and minimal N-dependent kinase activities were revealed. Hereto, the nutrient-rich growth medium of 3-day old PSB-D cell cultures (t −48h) was replaced by medium without N for 48 h to deplete the cells of internally available N reserves. During this 48 h N starvation period, we observed repression of TOR activity as the TOR-dependent S6K phosphorylation band gradually diminished over time and disappeared after 48h of starvation (t 0) (Fig. 1*B* and Supplemental Fig. S2*B*). In contrast, SnRK1 was activated during this N starvation period, as witnessed by the transcriptional upregulation of the SnRK1 target genes *DARK-INDUCIBLE 1* (*DIN1*), *TREHALOSE-6-PHOSPHATE SYNTHASE 8* (*TPS8*) and *TPS9* (Fig. 1*B*, left panel). Moreover, the N starvation state of the cell culture upon 48 h depletion of N was further confirmed by assessing the transcriptional response of several N starvation marker genes previously identified *in planta* (60), validating both the transcriptional repression of LBD37 and AT5G26200, as well as the induction of AT5G66110 upon N depletion (Supplemental Fig. S2C).

After 48 h of N starvation (t 0), cells were supplied with 1 mM KNO_3_, which led to the rapid activation of TOR after 15 min, demonstrated by the re-appearance of the S6K phosphorylation band (Fig. 1*B*, middle panel). This phosphorylation band was both TOR- and N-dependent, as it was absent when cells were treated with KNO_3_ in the presence of the TOR inhibitor AZD8055 or when KCl was supplied instead of KNO_3_ (Fig. 1*B*, middle panel). Notably, the N-dependent activation of TOR in PSB-D cells was not restricted to KNO_3_, given that NH_4_Cl and glutamine were also able to activate TOR (Supplemental Fig. S2*D*), in accordance with earlier observations made in Arabidopsis seedlings (19). Next to the activation of the TOR kinase, also the primary nitrate response (PNR) was activated in PSB-D cells upon KNO_3_ application, as shown by the transcriptional induction of the PNR genes *NITRATE REDUCTASE 1* (*NIA1*) and *NITRITE REDUCTASE* (*NIR*), which peaked 45 min after KNO_3_ addition (Fig. 1*B*, right panel). These results clearly illustrate that PSB-D cells are still responsive to N after the 48 h N-starvation period.

Finally, to obtain a more direct view on rapid SnRK1 responses upon KNO_3_ repletion, the ASP-SPARK reporter was expressed in PSB-D cells, and cells were N-starved for 48 h and followed by live imaging for 15 min after KNO_3_ repletion. The SnRK1 activity rapidly decreased over the first minutes, with a minimum between 10 and 15 min after the addition of KNO_3_, while the SPARK signal in the control condition without KNO_3_ remained stable (Fig. 1*C*). This response resembles the N-responsiveness of SnRK1 observed with the ASP-SPARK reporter in the shoots.

Taken together, a robust N-starvation/KNO_3_-repletion assay was established, showing the synchronized responsiveness of TOR and SnRK1 to altered N levels in PSB-D cells, as well as activation of the PNR upon KNO_3_ repletion.

### Mapping of the SnRK1 and TOR Interactome at Different N Levels

**Fig. 2:**
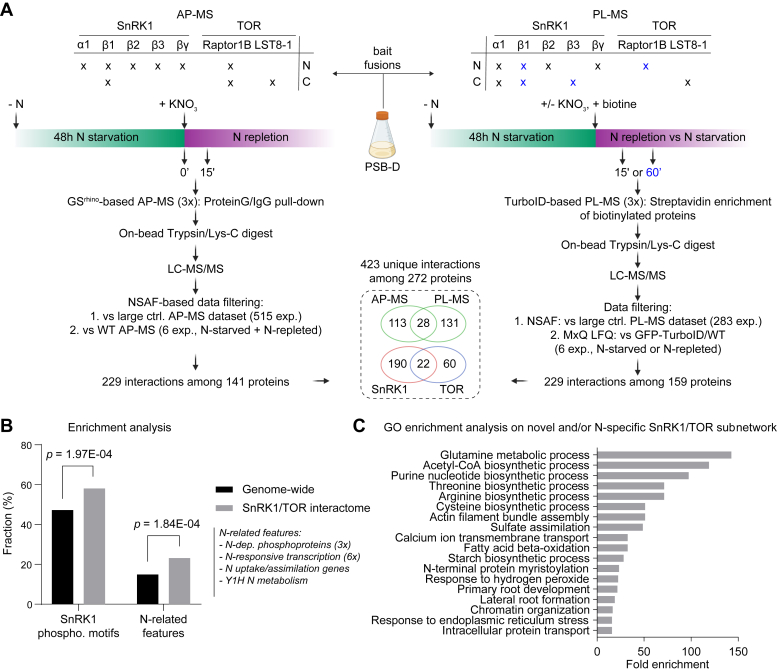
**Overview of the interactome mapping strategy and interactome enrichment analyses**. *A*, workflow of the AP-MS and PL-MS experiments that were performed to construct the interaction network, including a schematic diagram providing details on the bait fusion protein specifics (N = N-terminal fusion, C = C-terminal Fusion), points of sampling at time points zero (0′) and 15 min after N repletion for AP-MS, and T15 min (*black* (x)) or 60 min (*blue* (x)) with and without N repletion for PL-MS, the applied protocols for AP-MS and PL-MS, and strategies for data filtering. The number of specific and common interactors found with each kinase and the number of specific and common interactors for each method are summarized in the Venn diagram. *B*, enrichment analysis showing the fraction of proteins in the network that possess a SnRK1 phosphorylation consensus site or that are linked to N signaling, compared to the genome-wide fraction. *p*-values were calculated by the hypergeometric distribution test. *C*, gene Ontology (GO) enrichment analysis of biological processes enriched among the novel and/or N-specific subnetwork, excluding bait proteins. A selection of the most strongly enriched GO terms is shown.

To obtain a comprehensive view of how plant cells might signal N levels through TOR and SnRK1 and how these kinases translate this information into adequate growth responses, we performed a dynamic interactome screen, mapping interactors of both heterotrimeric kinase complexes in PSB-D cells, treated under the conditions established in our N-starvation/KNO_3_-repletion assay (Fig. 2*A*). For increased sensitivity during interactome screening, we used a combination of AP-MS and PL-MS. Based on knowledge from our prior interactome screens on TOR and SnRK1 (36, 42), multiple subunits of both protein kinase complexes were N and/or C-terminally fused to the GS^rhino^-tag for AP-MS (49) or to the TurboID-tag for PL-MS (50). Bait fusion proteins were constitutively expressed in PSB-D cell cultures (Supplemental Fig. S1), driven by the 35S promoter. These stably transformed cultures were harvested after 48h of N starvation and after KNO_3_-repletion, as depicted in Figure 2*A*.

In total, we performed interactomics experiments in triplicate for nine GS^rhino^ and nine TurboID bait fusion proteins, each under N-starved and KNO_3_-repleted conditions, together with corresponding negative controls (WT or 35S:GFP-TurboID). For AP-MS, protein complexes were purified under near-physiological conditions based on the high-affinity interaction between the Protein G moiety of the GS^rhino^-tag and Immunoglobulin G antibodies immobilized on magnetic beads. During PL-MS, proteins in the proximity of the bait fusion protein were *in vivo* biotinylated by the TurboID tag after treatment with 50 μM biotin for 15 min or 1 h. Next, biotinylated proteins were extracted and enriched under denaturing conditions through streptavidin binding. Proteins enriched by AP-MS or PL-MS were on-bead digested with Trypsin/Lys-C, analyzed through LC-MS/MS, and identified by Mascot and/or MaxQuant analysis.

For stringent identification of bait-specific protein–protein interactions (PPIs) from the resulting protein lists (Supplemental Table S2), a dual filtering strategy was applied for both interactomics methods (see Experimental Procedures and Fig. 2*A*). For the AP-MS samples, proteins identified by Mascot analysis were first compared to a large in-house AP-MS dataset. In this approach, averaged NSAF values were calculated for each identified protein and NSAF ratios were determined by comparing the bait experiments *versus* the large control AP-MS dataset. The latter dataset was generated in-house by 515 AP-MS experiments on 109 bait proteins not related to TOR, SnRK1, or N signaling. To control for potential proteome changes induced by the N-starvation/KNO_3_-repletion treatments, additional filtering was performed comparing NSAF values between the bait experiments and negative control AP-MS experiments on WT PSB-D cell cultures harvested under the same N-dependent conditions. Finally, specific PPIs were retrieved if they were retained with both filtering strategies (Supplemental Fig. S3*A*), revealing 229 interactions among 141 proteins (Supplemental Table S3*A*). For the PL experiments, proteins were identified through the integration of both Mascot and MaxQuant analyses. In the first phase, bait-specific interactors were highlighted by applying an NSAF-based large dataset filtering strategy on the protein lists resulting from the Mascot analyses, comparing against 283 PL-MS experiments on 37 bait proteins unrelated to TOR, SnRK1, or N signaling. To correct for potential background proteins induced during the N-starvation/KNO_3_-repletion assay, LFQ values of the MaxQuant-identified protein lists were compared to negative control PL-MS experiments on WT or 35S: GFP-TurboID cultures harvested under the corresponding N-starvation or KNO_3_-repletion regime (Supplemental Fig. S3*B*). Finally, both filtering strategies were integrated, giving rise to an interactome of 229 interactions among 159 proteins (Supplemental Table S3*B*).

### The N-Related SnRK1 and TOR Interactome Contains Many Known Interactors

After filtering of non-specific background proteins, the AP-MS and PL-MS subnetworks were combined, giving rise to a final interactome consisting of 423 interactions among 272 proteins (Supplemental Table S3, *C* and *D*). Confident interactions can be inferred by looking at interactions that were found under multiple experimental conditions, for instance with both AP-MS and PL-MS, with both the N- and C-terminal bait fusion protein, or under N-starved as well as KNO_3_-repleted conditions. As such, more than half of the interactions (239 out of 423) were independently confirmed, highlighting the quality of the obtained network. Furthermore, almost one-third of the proteins (80 out of 272) were found with multiple baits of the same kinase complex, adding further confidence to their corresponding interactions. Finally, interactions can also be evaluated through the Affinity Enrichment Score (Supplemental Table S3, *A* and *B*), which integrates both the fold enrichment (NSAF ratio) as well as the significance of the enrichment (Student’s *t* test *p*-value). As such, proteins can be ranked to pinpoint the strongest and/or most specifically enriched interactors. Typically, interactions with a high Affinity Enrichment Score represent stable interactions, involving for instance associations between the different core subunits of the kinase complexes, or with known SnRK1 regulatory proteins such as the class II TPS-like proteins, the FLZ proteins and the SnRK1-interacting negative regulator (SKIN) proteins (61).

When comparing AP-MS and PL-MS, only a limited overlap (35 interactions among 28 proteins) was found between both methods, in agreement with previous findings (36, 42). This low overlap is linked to the intrinsic technical specificities of both methods, which is why they were used as complementary discovery techniques in this study. When comparing both kinase complexes, the TOR complex accounts for 82 of the interactors in the network, while the more sampled SnRK1 complex yielded 212 interactors, with 22 proteins shared between both complexes. RAPTOR1B, a known SnRK1 substrate (28), was co-purified with multiple SnRK1 bait proteins, confirming the known crosstalk between TOR and SnRK1. To screen the network for other putative SnRK1 substrates, we indicated all proteins that harbor one or more SnRK1 consensus phosphorylation motifs (53) in their protein sequence (Supplemental Table S3*E*). Notably, this motif was significantly overrepresented in our interactome dataset (*p*-value = 1,97E-04, hypergeometric distribution), indicating that the network is enriched for *bona fide* SnRK1 substrates (Fig. 2*B*).

Given that proteins functioning within the same complex/pathway often tend to be transcriptionally co-regulated, the underlying transcriptional co-expression network of the interactome was investigated (55). This analysis revealed a dense co-expression network, connecting 56% of the interactome components (Supplemental Table S3*F* and Supplemental Fig. S4) and highlighting genes that are strongly co-expressed with SnRK1 or TOR complex subunits. For instance, strong co-expression was found between pantothenate kinase 2 (PANK2) and SnRK1α2, or between RAPTOR1B and PTEN2A, a homolog of human PTEN that acts upstream of mTORC1 signaling (62).

### Zooming in On the Relation of SnRK1 and TOR with N Signaling

**Fig. 3:**
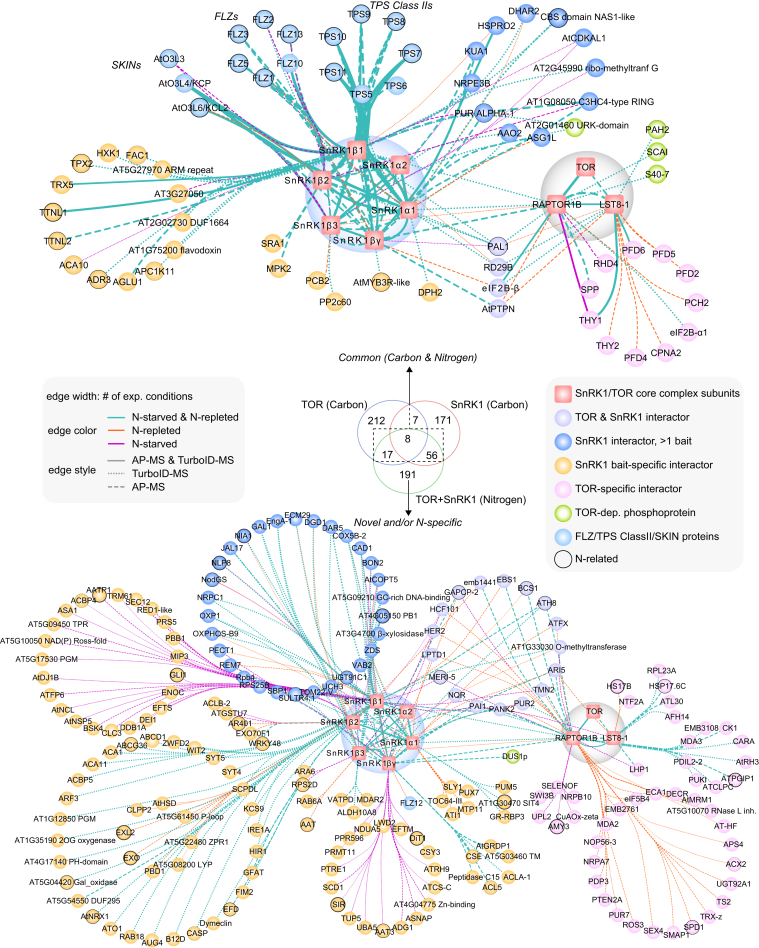
**Network visualization of the integrated interactome. Cytoscape visualization of the N-related SnRK1 and TOR interactome combining all AP-MS and PL-MS experiments**. Integrated node and edge attributes (Supplemental Table S3, *C* and *D*) are shown next to the network. The network was divided in a subnetwork covering interactions found in prior corresponding networks related to C signaling (*upper*), and a subnetwork covering all novel and/or N-specific interactions (*bottom*). Overlap with prior C-dependent TOR and SnRK1 interactome networks is shown in the Venn diagram. Nodes were circled as N related on the condition that the proteins were present in either N-related transcriptome or phosphoproteome datasets, a Yeast-one-Hybrid (Y1H) network for nitrogen-associated metabolism, or are known to be involved in N transportation (Supplemental Table S3*G*).

To reveal proteins that specifically interact with SnRK1 and/or TOR during N starvation and/or KNO_3_ repletion, we first compared the retrieved network to the corresponding C-related SnRK1 and TOR networks previously mapped in PSB-D cells upon sucrose-dependent synchronization or under default growth conditions (Fig. 3) (36, 42). Remarkably, 191 out of the 272 proteins were not found before, underscoring the novelty of the N-related SnRK1 and TOR interactome. Most of these are interacting with one specific subunit of SnRK1 or TOR. Especially with SnRK1β1, a high number of subunit-specific interactors was retrieved, covering, for example, BRASSINOSTEROID-SIGNALING KINASE 4 (BSK4), a homolog of BSK3 that integrates plant hormone signaling with root foraging under low N conditions (63), while other BSK homologs are integrating brassinosteroid signals downstream of TOR (64), and RAB18, a RAB GTPase that was recently described to play a pivotal role in linking the C/N balance with autophagy regulation (65). Finally, the nitrate reductase NIA1 and the transcription factor NLP8, a regulator of the primary N response and a close homolog of NLP7 (38), were identified as SnRK1β1 interactors, hinting towards a key role of this specific SnRK1 β-subunit in N signaling.

On the other hand, 81 proteins were found in common between the N- and C-related networks, providing a view on more general regulators and substrates of TOR and SnRK1, confirming, for instance, the intricate connection between SnRK1 and FLZs, SKINs, and class II TPS-like proteins, or between the TOR complex, THYMIDYLATE SYNTHASE 1 (THY1), the eukaryotic initiation factor 2B (eIF2B) complex and the prefoldin complex. When interpreting the novelty of the N-specific subnetwork, some caution is needed because adjusted filtering parameters were applied compared to previous analyses, and the large AP-MS and PL-MS control datasets were updated with additional experiments. Moreover, no PL-MS experiments were performed previously on the TOR complex. When comparing this novel TOR PL-MS data to the TOR-dependent phosphoproteomics dataset (42), some TOR-dependent phosphoproteins were now also detected as interactors, pinpointing them as common TOR substrates, such as the phosphatidic acid phosphohydrolase PAH2, an ortholog of the human TOR substrate LIPIN (66), or an unknown DUF3550 protein that we named SCAI because of its homology to the human Suppressor of Cancer Cell Invasion protein (67).

In an attempt to further elucidate possible dynamic interactors in the signaling network upon changes in N status, quantitative comparisons between N-starved and KNO_3_-repleted conditions were performed using MaxQuant analysis (Supplemental Figs. S5 and S6). These statistical analyses show that most proteins were not dynamically changing between the N-starved and KNO_3_-repleted conditions. From the list of proteins that do appear to be dynamically interacting with the TOR or SnRK1 complexes (Supplemental Table S4), only two (MDA2 found with RAPTOR1B, and NLP8 found with SnRK1β1) were found in the list of prey proteins retained in the networks we described earlier, and only a few were found to show this pattern in more than one bait experiment, demonstrating that they do not represent robust interactions. NIN-like protein 8 (NLP8) was more abundantly detected under N-starved conditions using PL-MS on SnRK1β1. This observation agrees with the known regulation of its close homolog NLP7, which is degraded during N starvation in a SnRK1-phosphorylation-dependent manner (37, 38). These limited dynamics were also observed when considering the whole network, as more than half of the interactions (237 out of 423) were found in common and retained between both conditions (Supplemental Table S3*C*). Moreover, most of the PPIs that were specifically enriched in only one condition were also found in the other condition, but below the applied enrichment thresholds.

To further evaluate the N specificity of the novel SnRK1 and/or TOR interactors, we plotted their NSAF values detected in N-starved, KNO_3_-repleted, and control (*i.e.* sucrose-related) experiments (Supplemental Figs. S7 and S8), illustrating that the largest fraction of novel SnRK1 and/or TOR interactors was indeed specifically isolated under N-related conditions. Some were now also retained in the carbon dataset because a slight modification of the filtering rules was applied. For example, the AP2/B3-like transcriptional factor REPRODUCTIVE MERISTEM 7 (REM7) (68), which was identified with three different SnRK1 subunits (βγ, β1, β2) as bait, albeit always with only one unique tryptic peptide. In contrast to our previous SnRK1/TOR networks, one-peptide hits (Supplemental Table S2*C*) were now retained if they were highly significant, otherwise a bias was created excluding small proteins such as REM7. Other exceptions were found in the TOR-related PL-MS subnetwork, *e.g.* the formin homology protein 14 (AFH14) was found as a highly specific LST8-1 interactor both under N- as well as under sucrose-related conditions, possibly reflecting a general relation between TOR and the cytoskeleton (69).

To further explore the link of the interactome with N signaling, we next integrated various N-related published datasets, mainly covering N-dependent transcriptome and phosphoproteome analyses (Supplemental Table S3*G*). This integration not only validated proteins known to play a role in N-dependent TOR or SnRK1 signaling, such as the N-related SnRK1 substrate NIA1 (43) and NLP8 (37, 38), but also revealed many interesting novel links. For instance, transcription of TTNL1, a disease resistance TIR-NBS-LRR protein that specifically interacts with SnRK1β1 under sucrose- and N-related conditions, is highly nitrate-dependent, in accordance with the recently discovered link between plant immunity and N status (70). Intriguingly, several class II TPS-like and FLZ proteins, which are mainly known to regulate SnRK1 in response to C levels (36, 71, 72), appear to be strongly regulated by N, pinpointing them as potential integrators that function in the crosstalk between N and C signaling. Similarly, the SnRK1 interactor we named NAS1 (AT3G52950, CBS domain NAS1-like), a homolog of GmNAS1, which functions as energy sensor in *Glycine max* root nodules during N fixation (73), is transcriptionally regulated by nitrate and might thus play a role at the interplay of SnRK1 with N and energy levels.

Overall, the N-dependent TOR and SnRK1 interactome was significantly enriched for genes harboring one or more of these N-related features (*p*-value = 1,84E-04, hypergeometric distribution) (Fig. 2*B*). This was further corroborated through a Gene Ontology (GO) analysis evaluating which biological processes were enriched in our network (Fig. 2*C* and Supplemental Table S3*H*). To correct for GO terms linked to the inherent role of SnRK1 and TOR related to C metabolism and sugar signaling, all TOR and SnRK1 core subunits as well as the subnetwork found in common between N and C were removed from the analysis. Among the N-related subnetwork, the glutamine metabolic process, which acts at the interface of C and N signaling (74, 75), and a known downstream process of TOR signaling in yeast (20), were the most significantly enriched. This observation is in agreement with the known function of TOR in regulating ammonium assimilation and glutamine metabolism (76), and further demonstrates the enrichment of N-related features present in the network. In general, many links with primary metabolism were found, as witnessed by the specific enrichment of proteins involved in the biosynthesis of primary metabolites, such as amino acids, acetyl-CoA, nucleotides, and starch, in fatty acid β-oxidation, or sulfate assimilation, covering thus typical processes that are tightly regulated by nutrient levels (77, 78, 79, 80). In addition, several novel relations were uncovered, for example, the link of SnRK1 with the master regulator of ER stress responses IRE1 (81) is largely understudied.

### Validation of Novel SnRK1 Substrates by *In Vitro* Kinase Assays

**Fig. 4:**
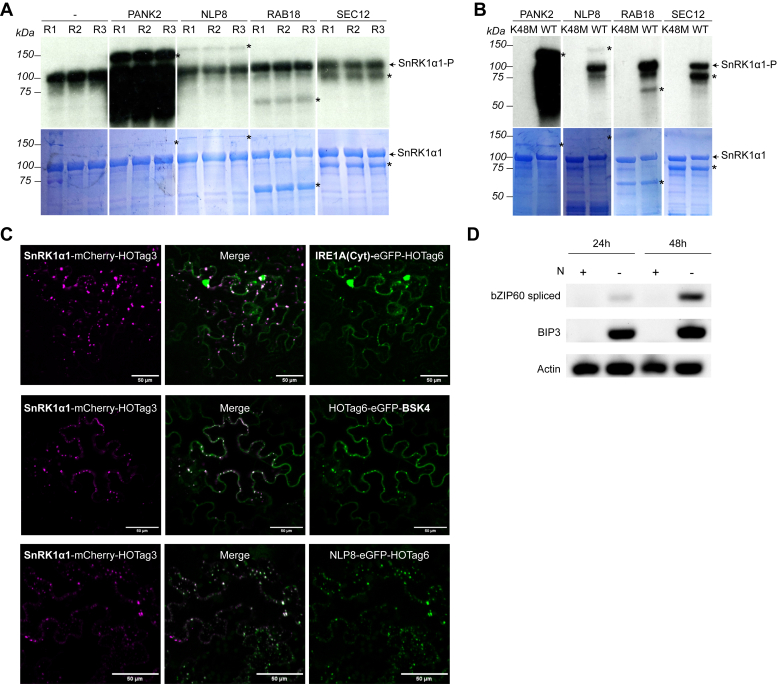
**Validation of a selection of novel N-related interactions**. *A* and *B,* autoradiograms and Coomassie brilliant blue R loading controls of *in vitro* kinase assays, confirming phosphorylation of recombinant PANK2 (142 kDa), NLP8 (146 kDa), RAB18 (65 kDa) and SEC12 (84 kDa) proteins N-terminally fused to a HisMBP tag by SnRK1α1 in the presence of ATP-γ-^32^P. Three experimental repeats are shown in (*A*) (R1-3), including a negative control without substrate addition (−). The negative controls with kinase-dead SnRK1α1 K48M are shown in (*B*). SnRK1α1-P corresponds to autophosphorylation of SnRK1α1, and substrate-specific phosphorylation bands are indicated with an asterisk. *C*, *in vivo* SPPIER-dependent validation of PPIs upon transient co-expression in *N. benthamiana* leaves of SnRK1α1-mScarlet-HOTag3 with IRE1A_cyt-eGFP-HOTag6, HOTag6-eGFP-BSK4, or NLP8-eGFP-HOTag6, as visualized by dual-colored phase-separated droplets. Scale bar, 50 μm. *D*, IRE1 activity assay. Five-day old seedlings were transferred to ½ MS medium containing either full N or no N and continued growing for the times indicated. RNA samples were analyzed for the presence of spliced bZIP60 and BiP3 mRNA by RT-PCR. Amplification with primers for actin mRNA was used as a control.

As a first mean to validate the novel and N-specific part of the interactome, putative SnRK1 substrates were recombinantly produced in *E. coli* to examine if they can be phosphorylated by SnRK1 in an *in vitro* kinase assay (Fig. 4, *A* and *B*). These proteins were selected based on the presence of the SnRK1 phosphorylation consensus motif (Supplemental Table S5*A*), their interaction profile, and/or a possible role in N signaling.

PANK2, involved in co-enzyme A biosynthesis (82), was selected because the protein harbors five SnRK1 phosphorylation motifs interacts with multiple subunits of the SnRK1 complex, and also interacts with the TOR complex. We could confirm its phosphorylation with a strong phosphorylation band induced by the catalytic SnRK1α1 subunit (Fig. 4*A*). Given the strong phosphorylation signal and the fact that PANK2 is a kinase, although it is not a protein kinase, control for potential autophosphorylation was included by usage of a kinase-dead (K48M) version of SnRK1α1, clearly demonstrating its SnRK1-dependent phosphorylation (Fig. 4*B*). Next, NLP8 was selected to further elaborate on the involvement of additional NLP proteins, next to NLP7, in SnRK1-mediated N sensing. In agreement with the presence of two SnRK1 phosphorylation motifs in NLP8, the observed phosphorylation band suggests that also NLP8 is a *bona fide* SnRK1 substrate (Fig. 4, *A* and *B*). The third protein, RAB18, which has one SnRK1 motif, was selected based on its recently discovered role in the integration of the C/N balance with autophagy (65), a well-described SnRK1-regulated process (83). The *in vitro* kinase assay supported that RAB18 is a putative SnRK1 substrate (Fig. 4, *A* and *B*), further adding it to the numerous downstream branches of SnRK1 that regulate autophagy. At last, also for the Sec12p-like protein (SEC12) SnRK1-dependent phosphorylation was observed (Fig. 4, *A* and *B*), in line with the presence of one SnRK1 consensus motif. This protein was selected because of its role at the ER as a GTP-exchange protein (84), hinting toward involvement in the trafficking of N-rich macromolecules, as well as given its homology to the *Saccharomyces cerevisiae* SEC12 protein, which is known to play a role in TOR signaling, autophagy regulation and amino acid sensing (84). Once these substrates were confirmed using radioactive kinase assays, similar non-radioactive kinase assays were performed (in duplicate, and including a control using a kinase-dead variant of SnRK1α1) and analyzed using MS to identify the corresponding phosphorylation sites (Supplemental Table S5*B*). For SEC12 (S184) and RAB18 (T99), the phosphorylated peptides with the highest intensity over the two repeats perfectly match with the SnRK1 consensus motif (53). For RAB18, one additional phosphorylation site was identified (S186), while for SEC12, seven additional phosphosites, specific for SnRK1α1 and not present in the SnRK1α1_K48M control samples, were identified (S166, S123, S101, S287, S226, S289, and S347 in order of intensity). These results confirm that SnRK1 can also phosphorylate non-consensus sites (13). For PANK2, 10 SnRK1 phosphorylation sites were identified (S286, T67, T521, S636, S124, S46, S321, T273, S509, and S323), three of which match with the SnRK1 consensus motif (S509, S46, and S286). For NLP8, five phosphosites were detected (T647, S101, S32, S160, and S162), however, none of them match with the known SnRK1 consensus motif. Similar observations were recently made for NLP7, where the identified SnRK1-dependent phosphosites S125 and S306 also do not match the consensus motif (38).

Finally, we also performed a radioactive *in vitro* kinase assay on the N-specific SnRK1 interactor BSK4. This protein does not contain a SnRK1 consensus motif, however, given that SnRK1 can also phosphorylate non-consensus sites, we assessed if BSK4 could be phosphorylated by SnRK1 (Supplemental Fig. S9). As no phosphorylation signal was observed, BSK4 is most probably not a direct SnRK1 substrate, suggesting that this protein could represent a novel upstream regulatory protein of SnRK1 which might integrate N status with plant hormone signaling, similar to the role of its homolog BSK3 (63).

### *In Vivo* Protein Interaction Validation of Candidate SnRK1 Regulators Using SPPIER

Some promising candidates from the network were chosen for a complementary interaction validation strategy. IRE1A, an endoribonuclease/protein kinase acting in ER stress and autophagy regulation (81), was identified as an N-specific interactor of SnRK1. Although this protein contains SnRK1 phosphorylation motifs, these motifs are located in its ER luminal domain, where SnRK1 is not presumed to operate (25), suggesting that IRE1A might not be a SnRK1 substrate. Notably, a recent paper described that the impairment of IRE1 function in an *ire1a/ire1b* double mutant leads to the hyperactivation of TOR, which could be closely linked to an altered IRE1-dependent regulation of SnRK1 activity (85). To further strengthen a possible link between IRE1A and SnRK1, *in vivo* interaction between IRE1A and SnRK1α1 was assessed using the SPPIER method (45), which allows efficient examination of PPIs upon transient expression in *N. benthamiana* leaves through visualization of dual-colored phase-separated droplets. As full-length IRE1A was not successfully expressed in this system, the cytosolic domain of IRE1A was selected and fused to eGFP and the homo-oligomerization tag 6 (HOTag6). Co-infiltration of this construct with the SnRK1α1-mCherry-HOTag3 fusion promoted the formation of dual-colored phase-separated droplets, pointing towards *in vivo* binary interaction between IRE1A and SnRK1α1 (Fig. 4*C*).

Next, the interaction was tested by SPPIER between BSK4 and SnRK1 to further support a possible direct relation between both proteins. Also here, dual-colored SPPIER droplets were observed when full-length BSK4, N-terminally fused to HOTag6-GFP, was co-infiltrated with SnRK1α1-mCherry-HOTag3 (Fig. 4*C*). Finally, also the interaction between SnRK1α1 and NLP8 could be visualized through SPPIER (Fig. 4*C*), not only strengthening its relationship with SnRK1-dependent N-signaling, but also demonstrating that transient kinase/substrate interactions can be investigated *in vivo* using the SPPIER method.

### The SnRK1-Dependent Regulation of N Starvation is Localized at the ER

In opisthokonts, N sensing by TOR and SnRK1 occurs at specific subcellular membrane components. In yeast, N sensing occurs on the membranes of vacuoles and endosomes (21), whilst in mammalian systems, this occurs on the membranes of lysosomes (22). As the structure of these endomembrane systems differs in plants, these kinases have likely evolved to be associated with another membrane-bound component. Intriguingly, multiple of the N-related and/or N-specific SnRK1 interactors identified in this study are linked to the ER, a known hub for TOR-dependent regulation of protein translation and regulation of SnRK1 activity (86), as demonstrated by the role of the ER as a platform for TOR and SnRK1 regulation by FLZ proteins (87). Furthermore, the novel interactor RAB18 was previously linked to the response to C and N starvation, as active RAB18 interacts with autophagy 18a (ATG18a) on the ER membrane (61). Additionally, SEC12 was identified as an N-specific interactor of SnRK1β1 with an ER localization that is involved in ER-to-Golgi transport (84) and was confirmed here by *in vitro* kinase assays as a substrate. Based on these interactions, the ER thus emerges as an important subcellular compartment where stress signals are integrated with protein synthesis *via* the tight regulation of translation. As a compartment that is at the center of stress responses and protein translation, a highly N-dependent process, the ER could be an integration spot for these signals.

The N-specific SnRK1 interactor IRE1A is an ER transmembrane protein that has been described to induce autophagy (31), through a currently unknown mechanism, further demonstrating the link between N signaling, SnRK1, and the ER membrane. The IRE1 proteins are dual kinases and endoribonucleases that act as sensors of the unfolded protein response (UPR), which is a stress pathway induced by the incomplete folding of proteins in the ER lumen (81). IRE1B is responsible for the alternative splicing of the mRNA that encodes bZIP60, a transcription factor featuring a basic leucine-zipper domain, crucial for the UPR in plants. The bZIP60 isoform encoded by the unspliced mRNA is retained in the cytosol, while spliced mRNA encodes a protein that can translocate to the nucleus, where bZIP60 plays a vital role in the activation of amongst others BINDING PROTEIN 3 (BiP3) during ER stress (88). Accordingly, detection of the spliced bZIP60 mRNA and induction of BiP3 can thus be used as a read-out for ER-stress and IRE1 activity (88). To further shed light on the role of IRE1 and the ER in N starvation, Arabidopsis seedlings were grown for 5 days on ½ MS, after which they were transferred to medium with or without N. Next, seedlings were harvested at 24 or 48 h and RT-PCR was performed monitoring spliced bZIP60 and BiP3 mRNA levels (Fig. 4*D*). Under normal growth conditions with sufficient N, no spliced bZIP60 or BiP3 induction could be observed. On medium without any N however, 24 h after the start of N starvation, spliced bZIP60 mRNA could be detected, and BiP3 expression was present, while after 48 h, this effect was even more pronounced. This observation demonstrates that in plants ER stress is induced by N starvation, and that IRE1 activation at the ER occurs under the same condition of N starvation that activates SnRK1. Moreover, our data show that IRE1 and SnRK1 show physical interaction, suggesting that they are functionally related in the process N signaling at the ER.

## Discussion

In this study, we mapped the SnRK1 and TOR interactome associated with N signaling. By comparing to corresponding networks previously mapped in the context of C signaling, we were able to highlight N-specific interactors. Through multi-omics N-dependent data integration, we could pinpoint a diverse set of proteins that might be related to N signaling, acting either up- or downstream of SnRK1 and/or TOR. Moreover, by merging the C/N-specificity analysis with the N-dependent data integration, we revealed novel candidate proteins such as the class II TPS-like and FLZ proteins that might integrate N with C levels through the regulation of SnRK1 and/or TOR.

Given that nutrients need to be absorbed by plants *via* roots and then transported for assimilation in either roots or shoots, some caution is needed when interpreting the N-related SnRK1/TOR interactome as this network was mapped in cultured cells and thus may not perfectly translate to whole plants and organs. Moreover, various cell types within the plant might respond differently to N signals. This was demonstrated by the SnRK1 ASP SPARK assay, where under N limitation, SnRK1 displays differential activity in roots and shoots. The attenuation of SnRK1 activity in N-starved roots is likely linked with an increase in TOR activity to promote primary root growth and elongation during the N-foraging response. This observation is in agreement with earlier observations showing that TOR activity in roots remains relatively high in N-starved conditions, whereas in the shoot TOR activity gradually decreases during N-starved conditions (19). Nonetheless, it might be that roots are not fully N-deprived under these starvation conditions due to nutrient re-allocation, while the N starvation assay was designed to show the response of completely N-deprived cells. It therefore makes sense for PSB-D cells to mainly resemble the shoot responses, showing an increase in SnRK1 and a gradual decrease in TOR activity during the 48 h N starvation period.

The difficulties with the identification of dynamic N-dependent interactors might be linked to the rapid and transient nature of N-dependent signaling events, their subcellular context, and the technical challenges associated with the identification of these highly dynamic interactions. The lack of dynamics demonstrates that it is currently still challenging to identify dynamic interactions by AP-MS and LP-MS, even in a homogenous system such as nutrient-synchronized cell cultures, in contrast with our prior phosphoproteomics analyses, which were more sensitive for the identification of dynamic sucrose-dependent phosphorylation events (36, 42). This might be linked to the transient and fast nature of the analyzed interactions involving the TOR or SnRK1 kinases. For instance, in human cells supplied with amino acids, the mTOR kinase is recruited to the lysosomes within 2 minutes, after which it is activated and leaves the lysosomes again within three to 4 min (89). Such fast biological responses face technical hurdles for interactomics. On the one hand, the PL TurboID enzyme needs a certain amount of time to accumulate sufficient levels of biotinylation on the interacting proteins for efficient enrichment. On the other hand, the spatial context and dynamics in subcellular transient signaling events can be lost in AP-MS during the *in vitro* cellular extraction of proteins. Furthermore, signaling events around *e.g.* ROP2 and TOR might be focused towards specific membrane-related subdomains, which are typically challenging to extract, separate, and analyze by AP-MS. The challenge to detect N-dependent dynamics at the protein level is also supported by an N-dependent phosphoproteome study (90) where only a limited number of differentially phosphorylated proteins was found. Finally, N signaling might be regulated in a more subtle manner than C signaling, or be strongly dependent on C signaling.

Remarkably, despite the lack of clear N-dependent dynamics, previous studies (19, 76) have shown, that nitrate and ammonium activate TOR directly and rapidly, whereas sugars tend to activate TOR more indirectly, for example *via* the repression of SnRK1 and thus de-repression of TOR activity, after its metabolization. However, how N is precisely sensed by SnRK1, and integrated in relation to C status, still needs further investigation. Nonetheless, as discussed below our data points to the ER as a potential regulatory hub where N status is integrated into SnRK1 and thus TOR signaling.

### The ER-Based SnRK1 Signaling Axis is Involved in Autophagy Regulation Under Low N Conditions

SnRK1 especially has a very dynamic subcellular localization (13, 83), with different model systems showing different subunits at various locations. SnRK1α1 is known to translocate to the nucleus under low energy stress (24), while under energy-rich conditions, it is localized at the ER (25). SnRK1β1 has been observed at the Golgi apparatus (84), whereas SnRK1β3 is believed to be the regulatory subunit localized in the nucleus (13). Integration of the C/N balance could be mediated by - amongst others - the FLZ and class II TPS proteins, which are transcriptionally N-regulated and also localized at the ER (36, 91). Furthermore, the ER is in direct contact with the nucleus, mitochondria, and chloroplasts, making it an extremely suitable hub for the exchange of energy and nutrient status signals between subcellular components.

In our PL-MS experiments with SnRK1β1, the discovery of many ER-localized interactions, such as IRE1A, RAB18, NLP8, and SEC12, points towards an ER-localized, N-dependent SnRK1 regulation. This pathway, which might be mediated by β1-specific SnRK1 complexes, appears to be spatially and functionally distinct from the mainly nuclear SnRK1α1 responses observed under sucrose starvation (36).

A cellular pathway that showed multiple links to SnRK1 in our network, especially in our validated set of interactors, is the regulation of autophagy, a process closely intertwined with both TOR and SnRK1 signaling (31). Proteins linked with the ER that have a clear role in the autophagic flux regulation are RAB18 and ATG8-interacting 1 (ATI1). In *RAB18* mutants, autophagy is compromised, especially under nutrient deprivation, affecting the ER association and expansion of ATG18a-positive autophagosomes (65). How RAB18 is activated under these starvation conditions is however poorly understood. As we observed both SnRK1-related interaction and *in vitro* phosphorylation of RAB18, this might suggest that active SnRK1 at the ER could regulate RAB18 under these conditions, possibly controlled by the IRE1-dependent activation of SnRK1.

Another autophagy-related protein from the interactome is ATI1, a SnRK1α1 interactor identified through AP-MS, that acts as an autophagy cargo receptor involved in reticulophagy and chlorophagy (92). Under favorable growth conditions, it is partially associated with the ER, whereas under starved conditions, it is present in ER and chloroplast-associated bodies, known as ATI-bodies, that are transported to the vacuole (92). Altogether, these data hint towards the existence of an ER-based autophagy-regulating pathway under the control of SnRK1 signaling.

Finally, the existence of this N-related pathway is further demonstrated by the PL-MS-based identification of IRE1A as an N-specific SnRK1β1 interactor. In Arabidopsis, IRE1B is required for reticulophagy, an important N-recycling process given that the ER is full of proteins in various stages of completion. However, its canonical downstream target, the transcription factor bZIP60 (84), is not required for IRE1B-dependent autophagy regulation, suggesting that the autophagy-regulating axis goes through a different pathway (31). Moreover, an ire1a/ire1b double mutant shows a TOR overexpression phenotype, corroborating a potential upstream function in SnRK1 signaling (81).

The role of IRE1 signaling in N sensing and signaling is currently not well established (88). Here, we demonstrated that stringent N starvation can induce IRE1-dependent bZIP60 splicing and BiP3 expression. This observation demonstrates that N starvation leads to ER stress, further demonstrating an intricate ER-based N starvation sensing pathway. Finally, the identification and confirmation of the interaction between the cytosolic domain of IRE1A and SnRK1 suggests that SnRK1, present at the ER, is involved in N signaling and possibly in autophagy induction. Further studies will be needed to elucidate the exact role of IRE1A and IRE1B in the regulation of SnRK1 activity under nitrogen stress.

### Engineering Opportunities

As N use efficiency is a valued agronomical trait, it is an interesting target for precision breeding of economically relevant crops. More insights into how this complex trait is regulated could therefore give rise to new engineering approaches. For example, it will be interesting to investigate N responses in single or higher-order knockouts of the specific class II TPS-like or FLZ proteins that are N responsive, as they might be important integrators of the C/N balance.

Another interesting protein discovered in this study with potential high economic value is the NAS1-like protein, which might couple N fixation with C status in legumes, being involved in the decision to invest valuable C sources into nodule formation (73). Its identification as a SnRK1 interactor in Arabidopsis might on the one hand reflect a broader function in the integration of energy and N levels in other plant species, while on the other hand, it will be valuable to study its association with SnRK1 in N fixation in nodules. Finally, the interactome shows that autophagy is a major downstream target for N-related SnRK1 signaling. Therefore, fine-tuning the regulation of this cellular recycling process through targets, such as RAB18 and ATI1, might lead to plants that are more resistant to limiting N conditions, such as in agricultural soil.

## Data Availability

The mass spectrometry interactomics and kinase assay data have been deposited to the ProteomeXchange Consortium *via* the PRIDE partner repository (93) with the dataset identifiers PXD054489 and 10.6019/PXD054489, and PXD054002 and 10.6019/PXD054002, respectively. Annotated spectra of the phosphorylated peptides from the kinase assays can be accessed through MS-Viewer (94) using the search key autzo6vlwr. The protein interactions from this publication have been submitted to the IMEx (http://www.imexconsortium.org) consortium through IntAct (95) and assigned the identifier IM-30002.

## Supplemental data

This article contains supplemental data (60, 90, 96, 97, 98, 99, 100, 101, 102, 103).

## Conflict of interests

The authors declare that they have no conflicts of interest with the contents of this article.

## Supplementary Data

Supplemental Table S5

Suppl. Figures
