# Supplementary material for: A Nitrogen-specific Interactome Analysis Sheds Light on the Role of the SnRK1 and TOR Kinases in Plant Nitrogen Signaling
Source: Mol Cell Proteomics. 2024 Sep 20;23(10):100842. doi: 10.1016/j.mcpro.2024.100842 (PMC11526089; doi:10.1016/j.mcpro.2024.100842)
Supplement: Suppl. Figures [file mmc6.pdf]

A

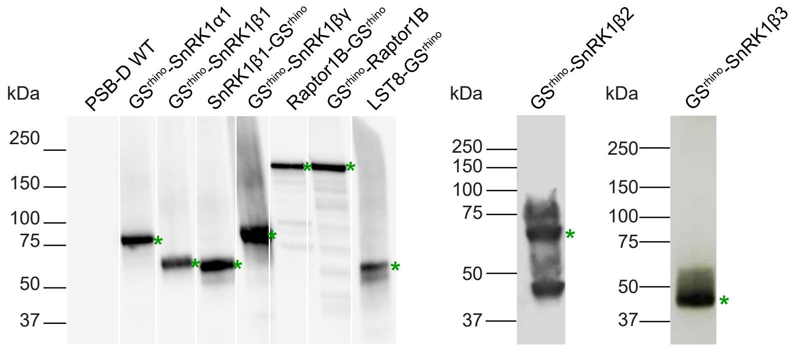

B

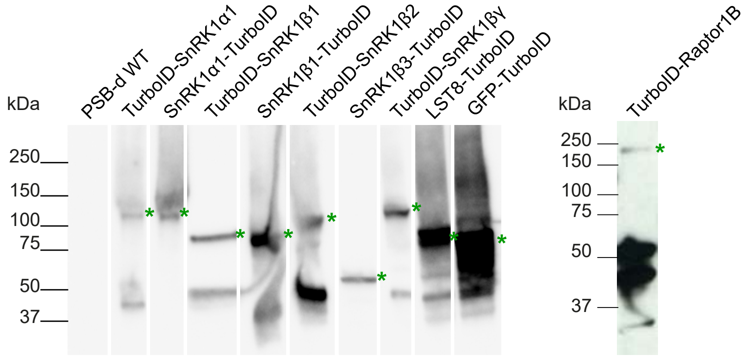

Supplemental Figure S1: Western blot expression analsis of GS^{rhino} and TurboID bait fusion proteins

(A) Expression analysis of GS^{rhino} bait fusion proteins by western blotting with peroxidase-anti-peroxidase (PAP) through affinity of the Protein G moiety in the GS^{rhino} tag for the anti-peroxidase antibodies in the PAP complex. As negative control, a total protein extract was analyzed of WT PSB-D cells. Full-length fusion proteins are indicated by a green asterisk.
(B) Expression analysis of TurboID-3xHA fusion proteins by western blotting with anti-BirA (mutated/TurboID) antibodies. As negative control, a total protein extract was analyzed of WT PSB-D cells. Full-length fusion proteins are indicated by a green asterisk.

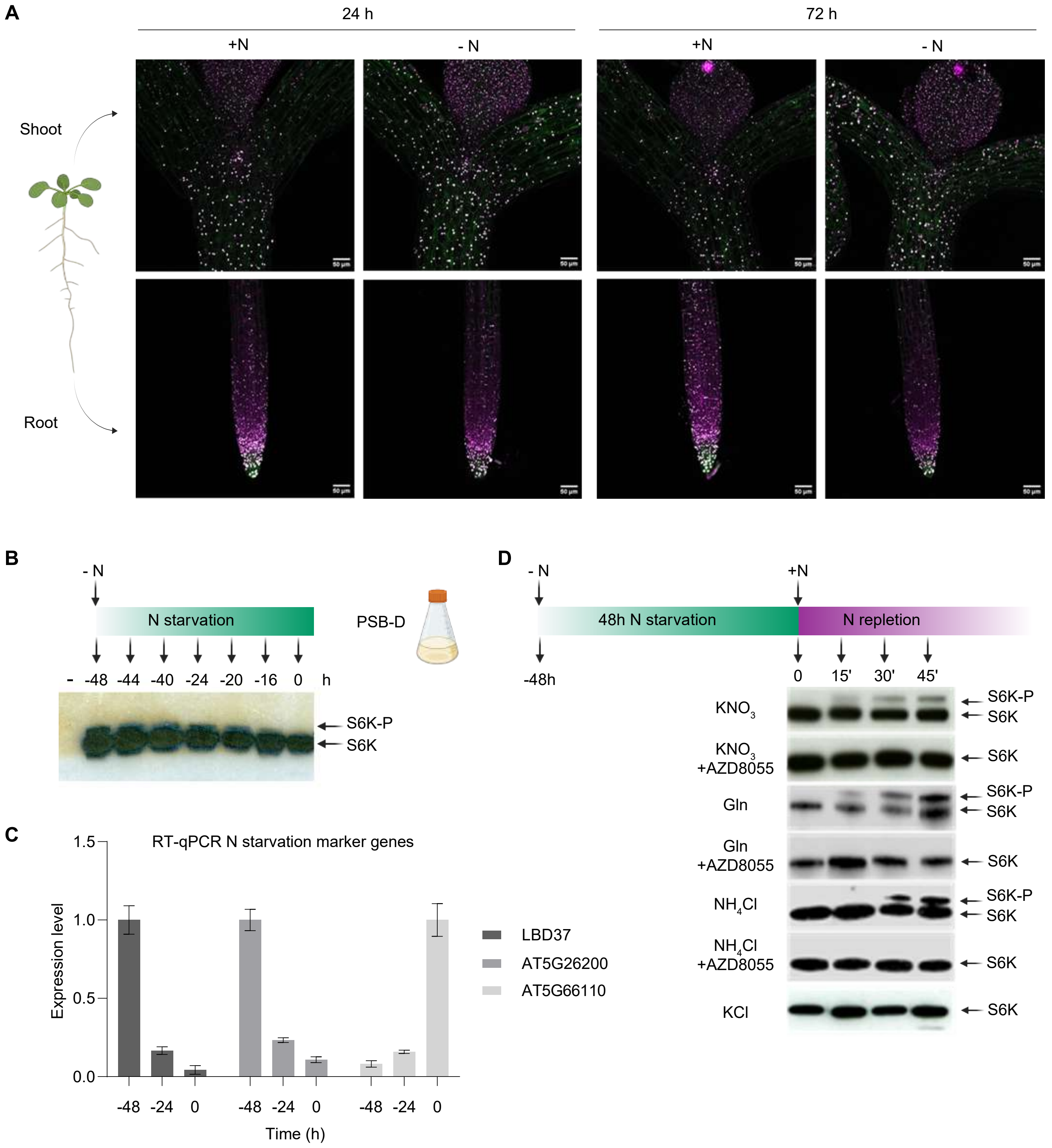

Supplemental Figure S2: Exploring N-dependent dynamics in SnRK1 and TOR activity in Arabidopsis.

(A) Visual representation of seedlings expressing the ASP-SPARK reporter for analysis of *in vivo* N-dependent SnRK1 kinase activity. Seedlings were grown on ½ MS without sucrose for five days, after which they were transferred to either fresh N-rich medium (+N) or medium without N (-N). Roots and shoots were imaged at either 24 h or 48 h after transfer.
(B) Anti-HA immunoblot analysis showing the influence of N starvation to cell cultures. A PSB-D cell culture expressing 35S:S6K1-3xHA was N-starved for 48 h, the phosphorylation status of S6K was analyzed at several timepoints. Bands corresponding to S6K and S6K-P are indicated with arrows. S6K-P represents the TOR-dependent phosphorylated isoform of S6K1-3xHA that migrates more slowly than its non-phosphorylated S6K isoform. As negative control (-), a total protein extract derived from WT PSB-D cell culture is shown.
(C) RT-qPCR results in PSB-D cells of three known N-starvation regulated genes, comparing transcript levels just before the initiation of the N depletion period (-48 h), and 24 h and 48 h after the start of the N depletion period. Transcript levels were normalized against two reference genes and against the maximal level of each gene.
(**D)** TOR activity read-out based on the phosphorylation of S6K via anti-HA immunoblot analysis. PSB-D cell culture expressing 35S:S6K1-3xHA was N-starved for 48 h, after which different N sources were added, with or without pre-incubation with the TOR inhibitor AZD8055. Activation of TOR activity was observed with 1 mM KNO_3, 1 mM NH_4Cl and 1 mM glutamine (Gln). In the KCl and AZD8055 controls, no activation of TOR was observed.

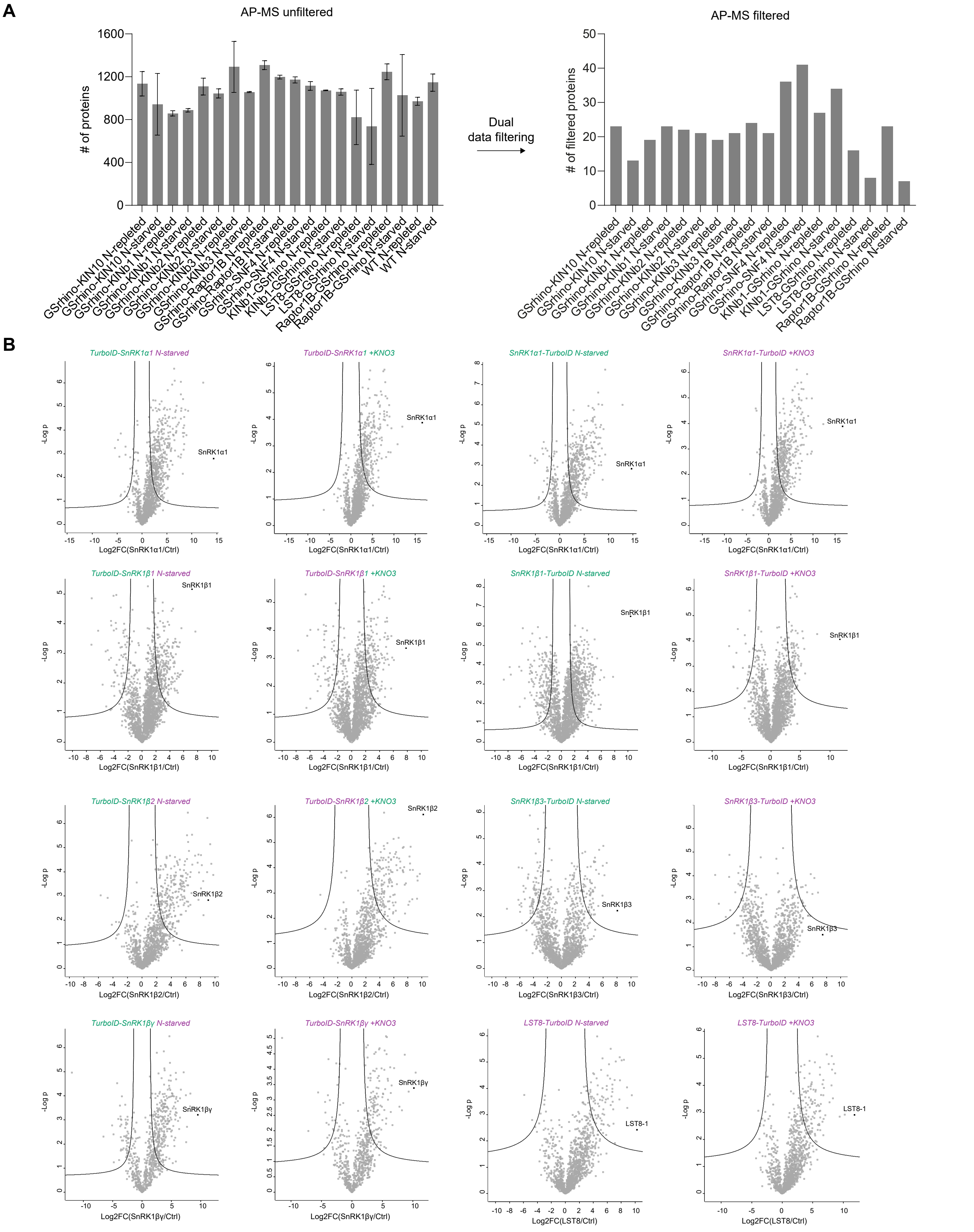

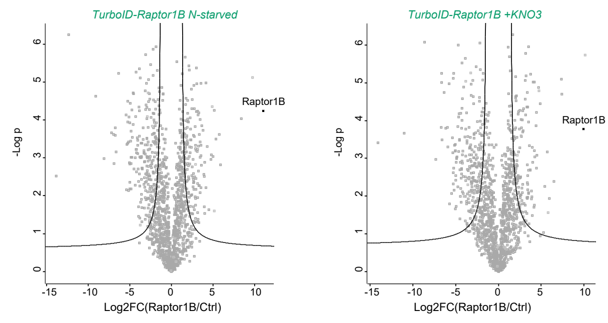

Supplemental Figure S3. Evaluation of background proteins in the interactomics dataset

(A) Histograms of Mascot-based AP-MS data showing the number of proteins before (left) and after (right) the dual filtering approach.
(B) Volcano plots of MaxQuant-based LFQ analysis on the PL-MS data.

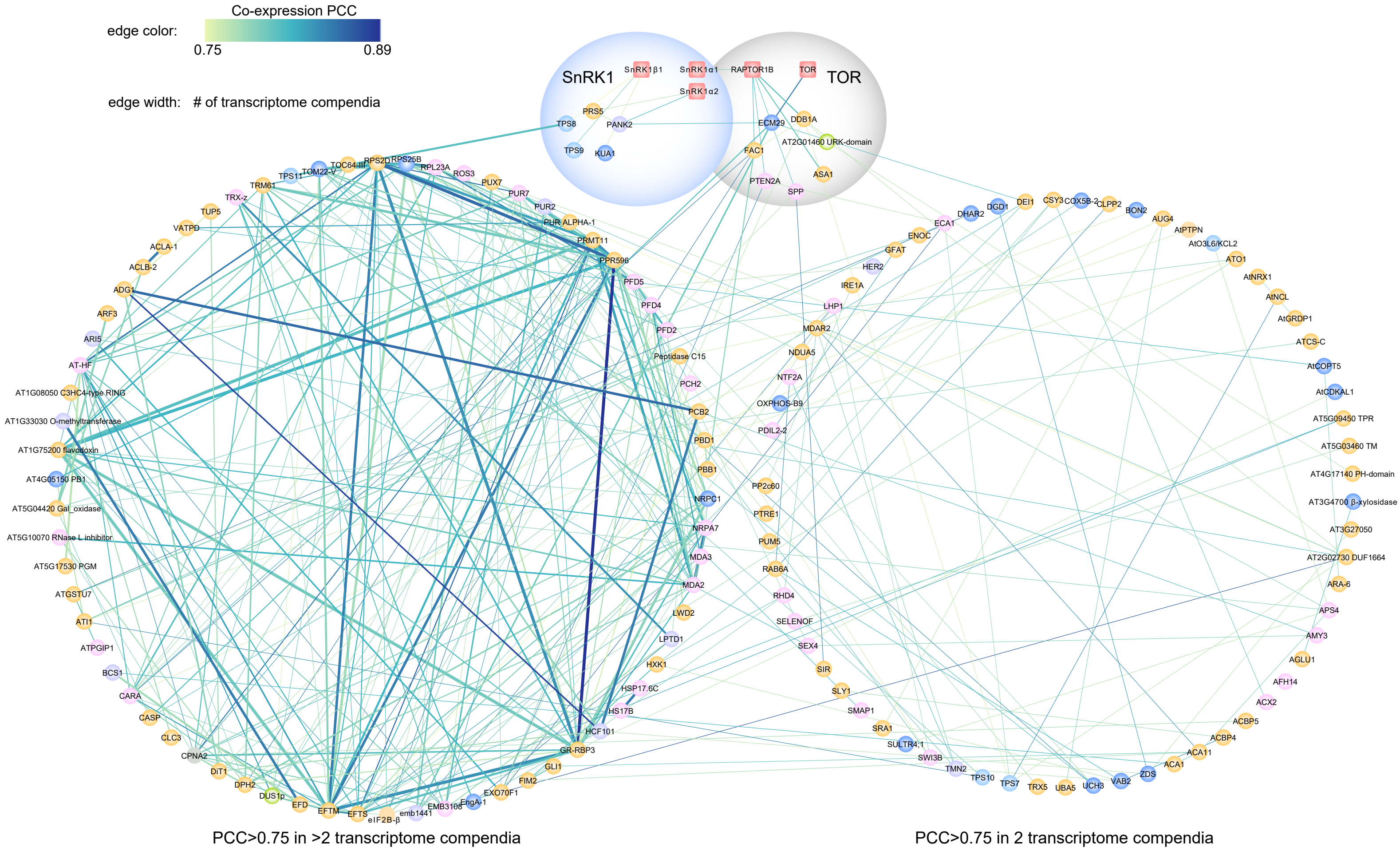

Supplemental Figure S4: Co-expression analysis on the N-dependent SnRK1 and TOR interactome

Cytoscape visualization representing the co-expression data from the Cornet analysis on the entire interactome network, covering interactors for which the corresponding genes are co-expressed with a Pearson Correlation Coefficient (PCC) of minimum 0.75, and present in at least two transcriptome compendia. Co-expression relations involving core SnRK1 and TOR subunits are shown on top. The co-expression network is further divided in two subnetworks involving co-expressing gene pairs in >2 (left) or =2 (right) transcriptome compendia. The edge color represents the PCC score, while the edge width shows in how many compendia the co-expression PCC was ≥ 0.75.

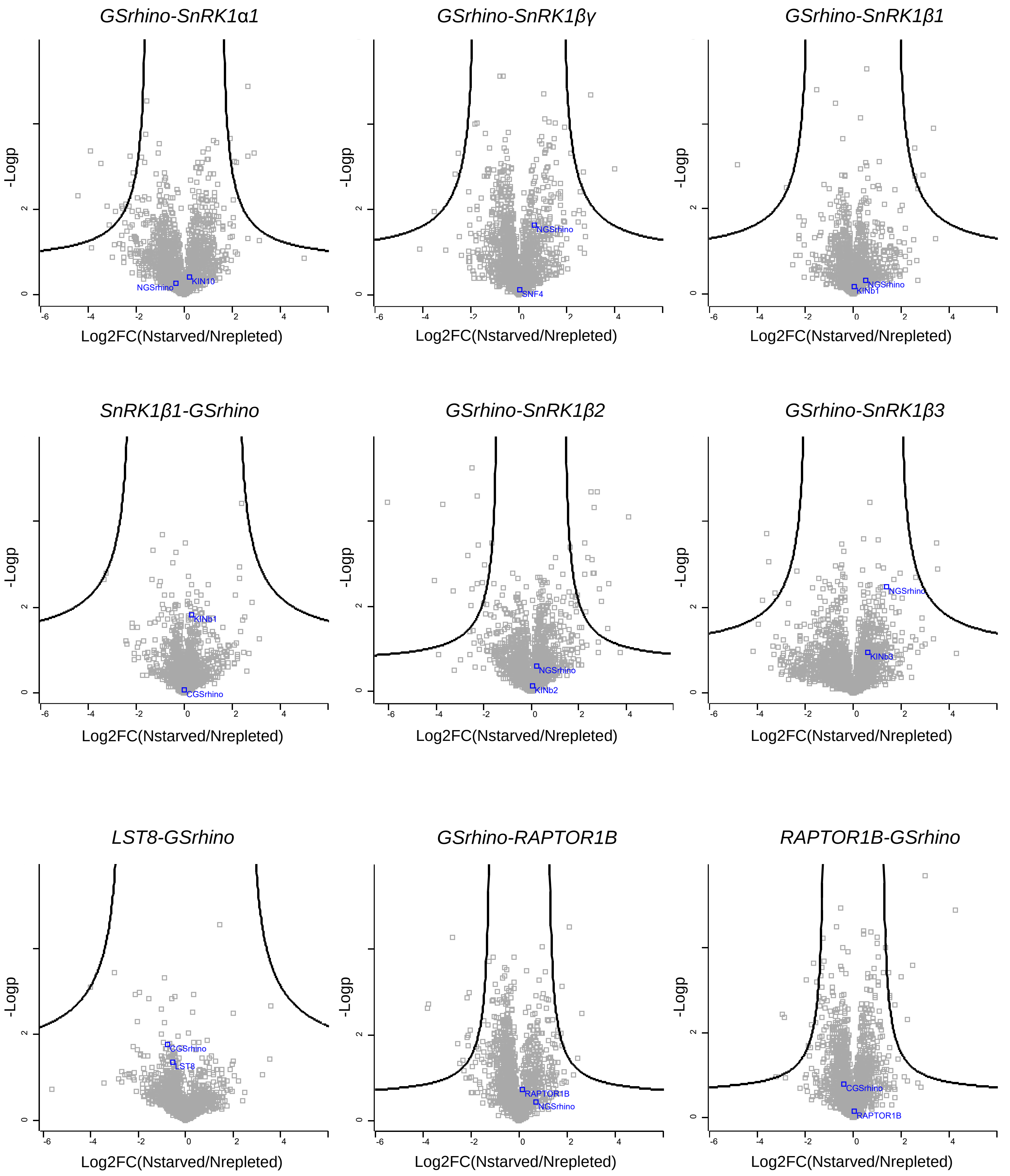

Supplemental Figure S5. MaxQuant LFQ-intensity based differential analysis between N-starved and KNO3-repleted conditions visualized in Volcano plots for AP-MS experiments

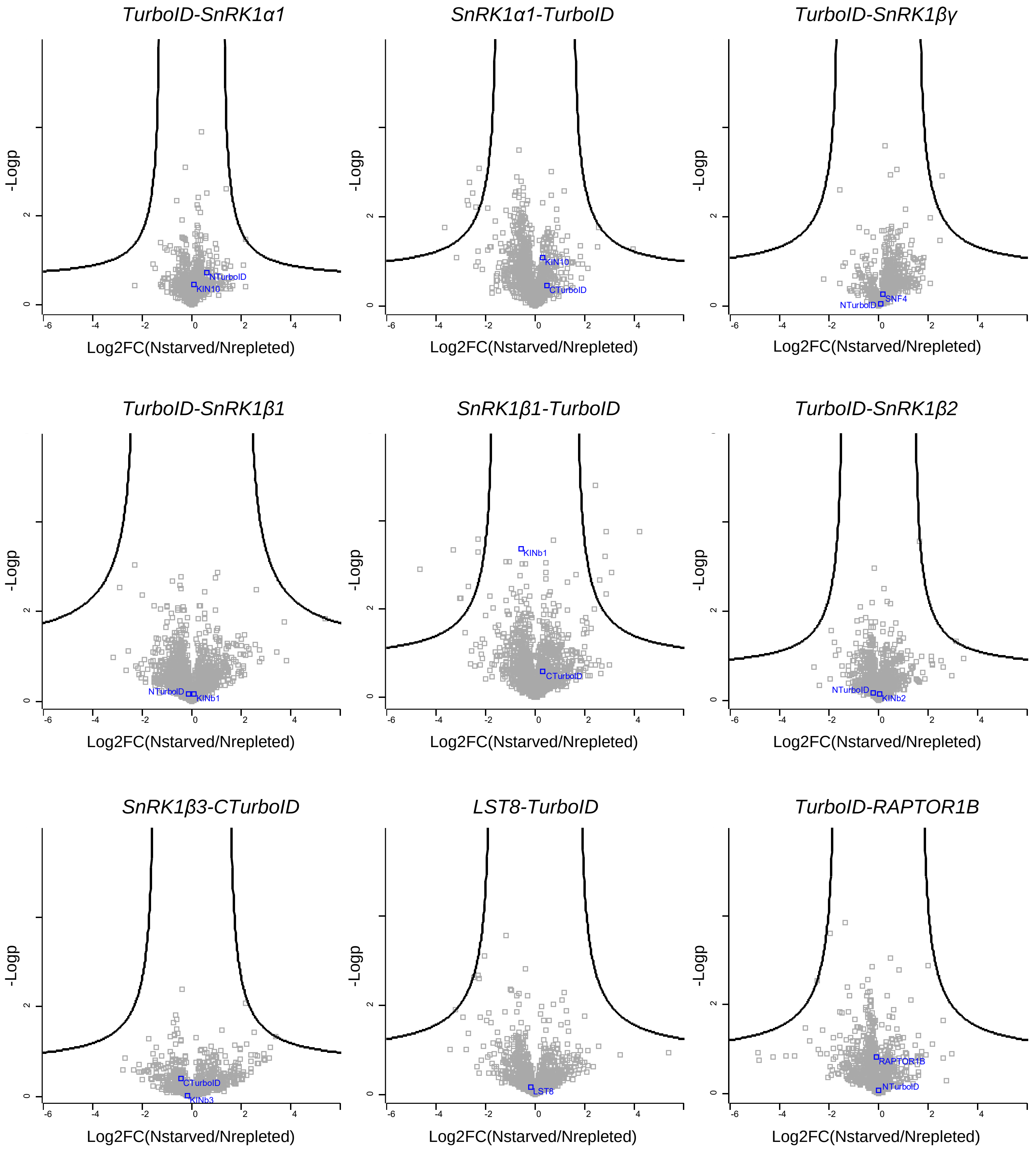

Supplemental Figure S6. MaxQuant LFQ-intensity based differential analysis between N-starved and KNO3-repleted conditions visualized in Volcano plots for PL-MS experiments

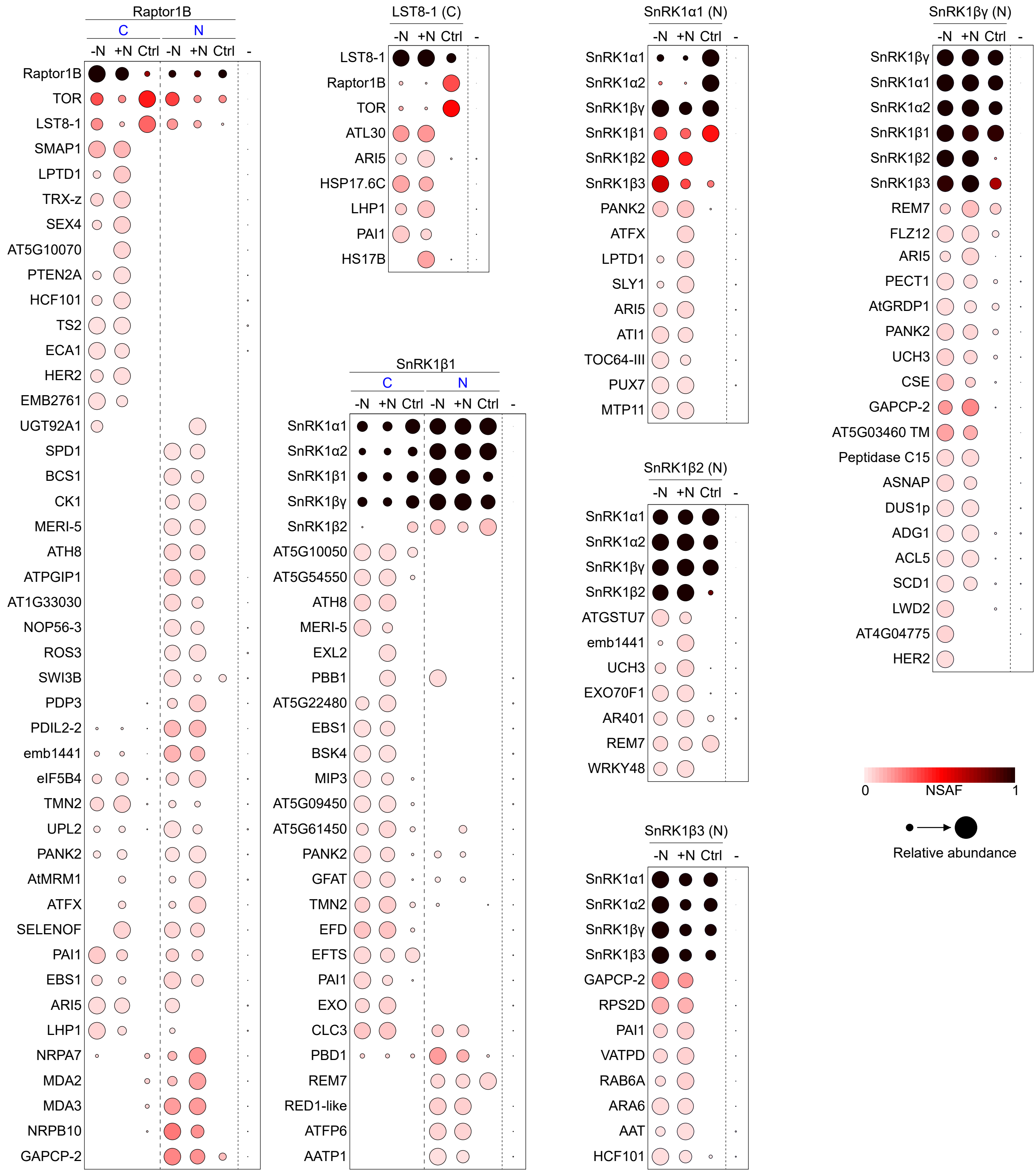

Supplementary Figure S7: N dynamics and specificity in the AP-MS data

Quantitative dot plot matrix representing the AP-MS data from the N-dependent TOR and SnRK1 interactome. The color of the nodes reflects the abundance of each prey in a given experiment, represented by the mean NSAF value. The size of the dots reflects the relative abundance of each prey over the different experiments. The terminus to which the tag was fused is indicated in blue (N- or C-terminus). Mean NSAF values in the corresponding (i.e. same bait fusion protein and method) carbon-related conditions (Ctrl) and negative (i.e. large control AP-MS dataset) control (-) are shown for comparison and evaluation of the N specificity. For coloring, NSAF values higher than 1 were capped at a value of 1.

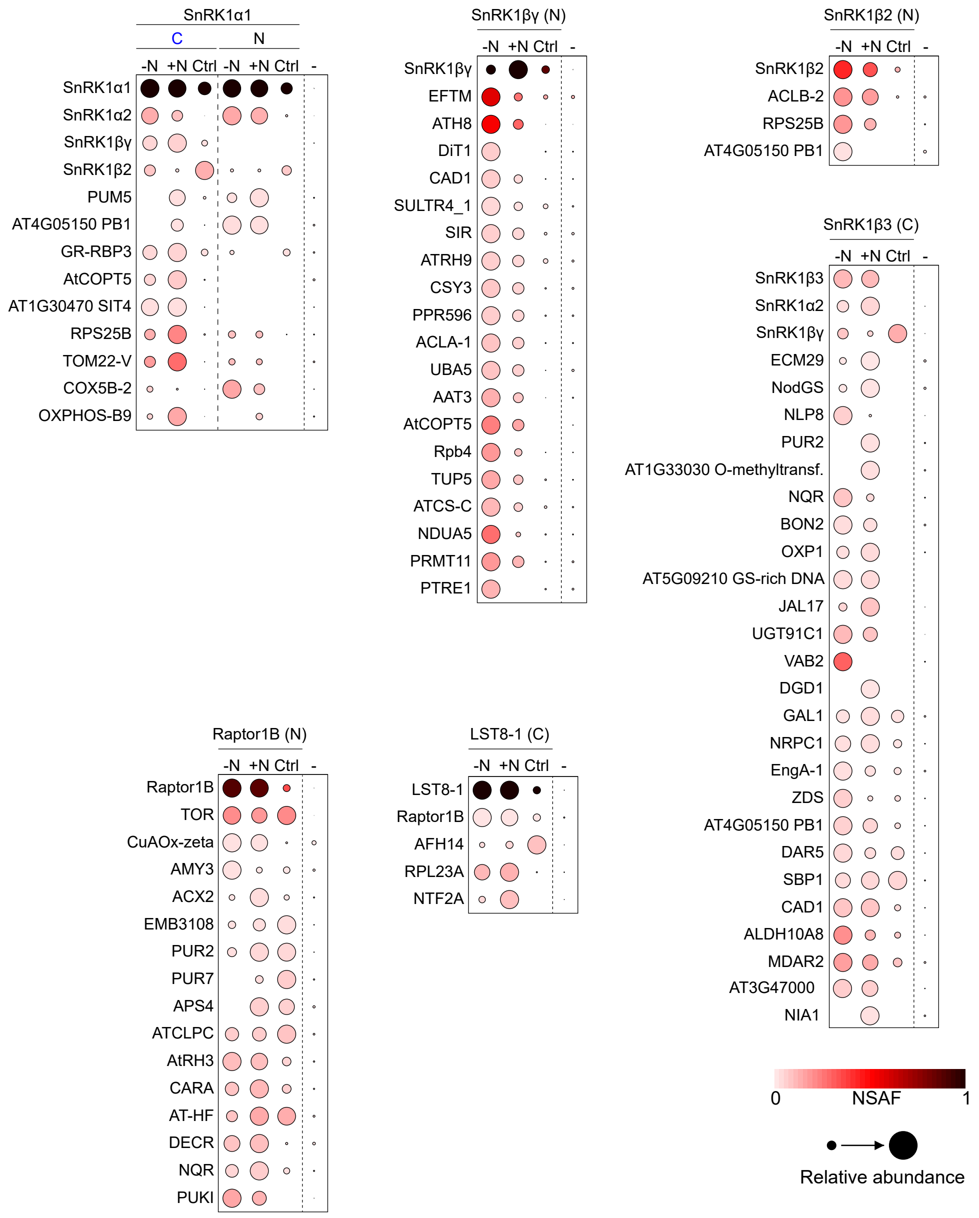

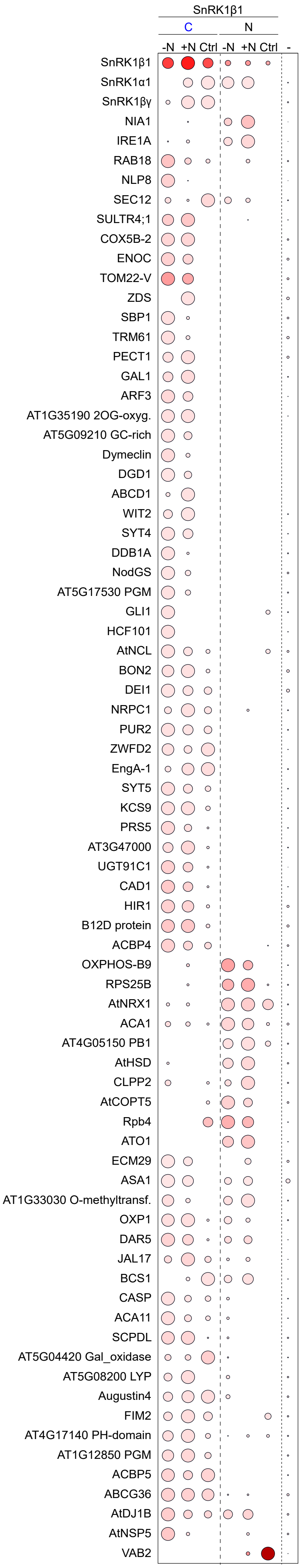

Supplementary Figure S8: N dynamics and specificity in the PL-MS data

Quantitative dot plot matrix representing the PL-MS data from the N-dependent TOR and SnRK1 interactome. The color of the nodes reflects the abundance of each prey in a given experiment, represented by the mean NSAF value. The size of the dots reflects the relative abundance of each prey over the different experiments. The terminus to which the tag was fused is indicated in blue (N- or C-terminus). Mean NSAF values in the corresponding (i.e. same bait fusion protein and method) carbon-related conditions (Ctrl) and negative (i.e. large control PL-MS dataset) control (-) are shown for comparison and evaluation of the N specificity. For coloring, NSAF values higher than 1 were capped at a value of 1.

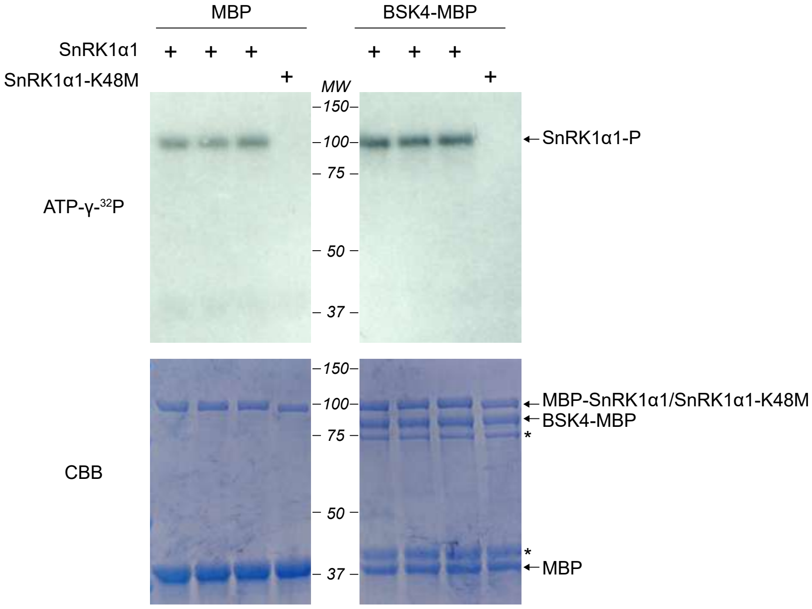

Supplemental Figure S9: Radioactive *in vitro* kinase assays showing BSK4 is not phosphorylated by SnRK1α1

Autoradiograms (ATP-γ-^{32}P) and Coomassie brilliant blue (CBB) R loading control of the *in vitro* BSK4 kinase assays. Three experimental repeats combining BSK4-MBP (95.5 kDa) with MBP-SnRK1α1 are shown (right panel), including triplicate corresponding negative controls with the MBP tag as such (41.5 kDa) added as substrate (left panel). As additional negative control, kinase-dead SnRK1α1 K48M was used. SnRK1α1-P corresponds to autophosphorylation of SnRK1α1, and BSK4-MBP degradation bands are indicated with an asterisk.
